# The Pathogenic Potential of *Campylobacter concisus* Strains Associated with Chronic Intestinal Diseases

**DOI:** 10.1371/journal.pone.0029045

**Published:** 2011-12-14

**Authors:** Nadeem O. Kaakoush, Nandan P. Deshpande, Marc R. Wilkins, Chew Gee Tan, Jose A. Burgos-Portugal, Mark J. Raftery, Andrew S. Day, Daniel A. Lemberg, Hazel Mitchell

**Affiliations:** 1 School of Biotechnology and Biomolecular Sciences, The University of New South Wales, Sydney, New South Wales, Australia; 2 Systems Biology Initiative, The University of New South Wales, Sydney, New South Wales, Australia; 3 Biological Mass Spectrometry Facility, The University of New South Wales, Sydney, New South Wales, Australia; 4 Department of Paediatrics, University of Otago (Christchurch), Christchurch, New Zealand; 5 Department of Gastroenterology, Sydney Children's Hospital, Sydney, New South Wales, Australia; Indian Institute of Science, India

## Abstract

*Campylobacter concisus* has garnered increasing attention due to its association with intestinal disease, thus, the pathogenic potential of strains isolated from different intestinal diseases was investigated. A method to isolate *C. concisus* was developed and the ability of eight strains from chronic and acute intestinal diseases to adhere to and invade intestinal epithelial cells was determined. Features associated with bacterial invasion were investigated using comparative genomic analyses and the effect of *C. concisus* on host protein expression was examined using proteomics. Our isolation method from intestinal biopsies resulted in the isolation of three *C. concisus* strains from children with Crohn's disease or chronic gastroenteritis. Four *C. concisus* strains from patients with chronic intestinal diseases can attach to and invade host cells using mechanisms such as chemoattraction to mucin, aggregation, flagellum-mediated attachment, “membrane ruffling”, cell penetration and damage. *C. concisus* strains isolated from patients with chronic intestinal diseases have significantly higher invasive potential than those from acute intestinal diseases. Investigation of the cause of this increased pathogenic potential revealed a plasmid to be responsible. 78 and 47 proteins were upregulated and downregulated in cells infected with *C. concisus*, respectively. Functional analysis of these proteins showed that *C. concisus* infection regulated processes related to interleukin-12 production, proteasome activation and NF-κB activation. Infection with all eight *C. concisus* strains resulted in host cells producing high levels of interleukin-12, however, only strains capable of invading host cells resulted in interferon-γ production as confirmed by ELISA. These findings considerably support the emergence of *C. concisus* as an intestinal pathogen, but more significantly, provide novel insights into the host immune response and an explanation for the heterogeneity observed in the outcome of *C. concisus* infection. Moreover, response to infection with invasive strains has substantial similarities to that observed in the inflamed mucosa of Crohn's disease patients.

## Introduction

The human host first comes in contact with a rich array of intestinal bacteria, both non-pathogenic and potentially pathogenic, at the surface of the thick mucus layer that covers the mucosal surface of the intestine. Under specific conditions, some of these bacteria can penetrate the mucus layer, adhere to and invade the mucosa, and subsequently cause chronic intestinal diseases.

Crohn's disease (CD) is one of two major types of inflammatory bowel diseases. It is a chronic, relapsing active inflammatory disease affecting any part of the human gastrointestinal tract. Currently, the major differential diagnosis of CD from acute and self-limited gastroenteritis relies upon the presence of particular pathological findings including acute and chronic inflammatory cell infiltrates, the branching of intestinal crypts, granulomata and remodelling of the epithelial layer as well as the presence of symptoms for several weeks and recurrent symptomatic bouts of disease [1], [2]. Despite much research over many decades, no consensus has been reached regarding its etiology, however, there is strong evidence to support the role of bacteria in this disease [3].

It has been postulated that mucosa-associated bacteria (MAB) due to their morphological and motility features may penetrate and break the mucus barrier, thus allowing them to adhere to, invade, and subsequently colonize the intestinal mucosa layer [4]. These MAB include the spiral-shaped *Campylobacter* species, many of which are equipped with corkscrew-like motion that allows them by means of their flagella to move through the mucus layer to the epithelial surface [5].

In 2009, Zhang *et al* [6] reported the molecular detection of *Campylobacter* species in biopsy samples of children with newly diagnosed CD and controls. Interestingly, *C. concisus* DNA was found to be significantly more prevalent in children with CD (51%) than in controls (2%). Importantly in this study, *C. concisus* UNSWCD was isolated from a child with CD, providing evidence that in the early stages of CD, viable *C. concisus* species are present in the intestinal tracts of CD children. In 2010, further support for the possible role of *C. concisus* in CD was provided in a study by Man *et al* who reported the prevalence of *C. concisus* to be significantly higher in fecal samples of CD children as compared with that in non-CD inflammatory and healthy control groups [7]. Studies on *C. concisus* UNSWCD showed that this strain had an increased ability to invade the intestinal cell line Caco-2 as compared with strains isolated from patients with acute gastroenteritis and healthy controls [8]. In addition, a range of virulence factors have been identified to be secreted by *C. concisus* UNSWCD, including a RTX toxin and an outer membrane fibronectin binding protein [9].

Given that the current literature suggests that *C. concisus* is genetically and taxonomically diverse [4], [10], further studies investigating whether other isolates from chronic intestinal diseases have similar invasive abilities as the UNSWCD strain were required. In this study, a method to isolate MAB from intestinal biopsies of patients with chronic intestinal diseases and healthy controls was developed and, the ability of eight *C. concisus* strains isolated from patients with chronic intestinal diseases (three of which were isolated in this study) to adhere to and invade intestinal epithelial cells was investigated. The feature that likely confers the invasive phenotype of *C. concisus* was elucidated. Furthermore, we examined the effect of *C. concisus* UNSWCD on the protein expression in the human intestinal epithelial cell-line, Caco-2, using two dimensional (2D) gel electrophoresis coupled with tandem mass spectrometry. The regulation of inflammatory pathways identified through proteomics were confirmed with ELISA.

## Materials and Methods

### Isolation of mucosa-associated bacteria from intestinal biopsies

A method to isolate MAB from intestinal biopsies based on a two-step enrichment-filtration procedure was developed. For the enrichment step, Ham's F-12 was employed as an enrichment broth as this medium had been reported to have the unique property of providing stable growth of *Helicobacter pylori* even without the addition of serum [11]. The second step involved filtration of the complex growth mixtures from the enrichment broth through size-specific porous membranes that allowed for the separation of highly motile MAB from other non-motile or less motile bacteria [12]. Mucosal biopsy specimens from symptomatic children undergoing colonoscopy at the Sydney Children's Hospital (Randwick, Australia) were collected from an area adjacent to areas of inflammation within the ileo-colonic region of the intestine. Biopsy specimens were enriched in 3 ml Ham's F-12 media (Invitrogen) containing 5% fetal bovine serum (FBS) and vancomycin (10 µg ml^−1^) for 48 h at 37°C after which 200 µl of the growth mixture was filtered through a 0.6 µm Whatman filter (Interpath Services) onto Horse Blood Agar containing vancomycin (10 µg ml^−1^) and incubated at 37°C under microaerobic conditions generated by a *Campylobacter* gas generating system (Oxoid) for a further 48 h. Colonies were visualized under phase contrast microscopy and near complete 16S rRNA gene sequencing using the primers F27 and R1494 [13] was performed on all colonies of interest (spiral morphology).

### Ethics approval

This study was approved by the Research Ethics Committees of the University of New South Wales and the South East Sydney Area Health Service-Eastern Section, Sydney (Ethics No.: 06/164). Written consent was obtained from all subjects, or their guardians, participating in this study.

### Bacterial species and strains, and growth conditions

*Campylobacter concisus* strains UNSWCD, UNSW1, UNSW2, UNSW3, ATCC 51561, ATCC 51562, UNSWCS and BAA-1457 were used in this study. All strains were grown on Horse Blood Agar (HBA) plates [Blood Agar Base No. 2 supplemented with 6% defibrinated horse blood (Oxoid)], and incubated at 37°C under microaerobic conditions for 48 h. *Salmonella* Typhimurium LT2 and *Escherichia coli* K-12 were grown on Nutrient agar (Oxoid) under atmospheric conditions at 37°C for 24 h.

### Cell culture

Three cell lines were used in this study, the human intestinal epithelial cell line Caco-2 (American Type Culture Collection; HTB-37), the human mucin producing intestinal cell line LS174T (American Type Culture Collection; CL-188) and the human monocytic leukemia THP-1 cell line (ATCC No.: TIB-202).

### Caco-2 cells

Cells were grown in 10 ml cell culture media comprised of Minimum Essential Medium (MEM), (Invitrogen) supplemented with 10% FBS, 1 mM sodium pyruvate, 0.1 mM non-essential amino acids, 2.25 mg 1^−1^ sodium bicarbonate and 100 µg ml^−1^ penicillin and streptomycin (Invitrogen) in 25 cm^2^ tissue culture flasks (In Vitro Technologies; Noble Park, VIC, Australia) at 37°C with 5% CO_2_. After 1 week of culture, cells were harvested by trypsinization. Cells were either passaged at a concentration of 1×10^5^ cells ml^−1^ into 25 cm^2^ tissue culture flasks and maintained for a week or seeded at a concentration of 5×10^5^ cells ml^−1^ into 24-well plates and kept for 2 days at 37°C with 5% CO_2_ in order to form a confluent monolayer for the adherence and invasion assays. Prior to seeding, the wells were coated with 1 ml collagen (0.338 mg ml^−1^) and incubated for 20 min at 37°C with 5% CO_2_.

### Intestinal cell line LS174T

Cells were grown in 10 ml cell culture media comprising Roswell Park Memorial Institute (RPMI)-1640 medium (Invitrogen) supplemented with 10% FBS and 100 µg ml^−1^ penicillin and streptomycin in 25 cm^2^ tissue culture flasks at 37°C with 5% CO_2_. After 2 days of culture, cells were harvested by trypsinization. Cells were then either passaged at a concentration of 5×10^5^ cells ml^−1^ into 25 cm^2^ tissue culture flasks and kept for 2 days or seeded at a concentration of 5×10^5^ cells ml^−1^ into 24-well plates and kept for 2 days to form a confluent monolayer. The confluent monolayer was incubated at 37°C with 5% CO_2_ for an extra 3 days to allow the development of a mucin layer for the adherence and invasion assays. The medium was changed daily until the development of a mucin layer.

### THP-1 cells

Cells were cultured in RPMI 1640 medium containing 2 mM L-glutamine (Invitrogen) supplemented with 10% FBS, 1 mM sodium pyruvate, 2.25 mg l^−1^ sodium bicarbonate and 100 U ml^−1^ penicillin and streptomycin. After 1 week of culture, cells were harvested by centrifugation. Cells were either passaged at a concentration of 2×10^5^ cells ml^−1^ into 25 cm^2^ tissue culture flasks and maintained for a week or seeded at a concentration of 2×10^5^ cells ml^−1^ with 250 nM phorbol 12-myristate 13-acetate (PMA) into 96-well plates to differentiate into macrophages. Following incubation for 2 days, the media with 250 nM PMA was replaced and the cells were used for ELISA assays the following day.

### Gentamicin protection (invasion) and adherence assays

Monolayers were infected with the bacteria at a Multiplicity of Infection (MOI) of 200. Following the addition of the bacteria, the 24-well plates were centrifuged at 232× *g* for 5 min to promote bacterial-human cell contact. Infected monolayers were then co-incubated with the bacteria for 6 h at 37°C with 5% CO_2_ to allow adherence and invasion to occur.

Invasion assays were performed as previously described by Man *et al* [8]. As *C. concisus* UNSW1 exhibited decreased sensitivity to gentamicin a modification was made where monolayers were treated with cell culture media containing 100 µg ml^−1^ penicillin and streptomycin plus 200 µg ml^−1^ gentamicin during the 1 h incubation to kill any extracellular bacteria.

For the adherence assays, the monolayers were washed four times with antibiotic-free cell culture media to remove extracellular bacteria, and were lysed with 0.5 ml 1% Triton X-100 for 5 min to release internalized bacteria. The lysate solutions from each monolayer were plated in quadruplicate on suitable media. All adherence assays were performed in duplicate and all experiments were repeated three times. Bacterial adherence was calculated by subtracting the internalized bacteria determined using the gentamicin protection assay from the bacterial counts obtained using the adherence assay, and expressed as a relative percentage of inoculated bacteria.

The statistical significance of the differences between the levels of adherence and invasion (mean ± standard deviation) achieved by the different strains of *C. concisus* was determined using the unpaired *t*-test using Prism GraphPad version 5.0 (GraphPad Software; San Diego, CA, USA).

### Antibiotic susceptibility testing

As gentamicin failed to kill all extracellular *C. concisus* UNSW1, the susceptibility of *C. concisus* UNSW1 to gentamicin was examined using the Epsilometer (E)-test system according to the manufacturer's instructions (AB Biodisk; Solna, Sweden). Based on the E-test, the minimum inhibitory concentration (MIC) of gentamicin required to inhibit *C. concisus* UNSW1 was 1.5 µg ml^−1^. Unfortunately, no adequate standard for gentamicin susceptibility testing for *Campylobacter* strains are available [14], and thus, we were unable to determine if *C. concisus* UNSW1 fell into the susceptible, intermediate or resistant category. Despite this, the MIC value for UNSW1 is considerably higher than that previously reported for other *C. concisus* strains (∼0.03 µg ml^−1^) examined by Vandenberg *et al* [15]. This reduced susceptibility is likely to explain the failure of gentamicin to successfully kill the extracellular *C. concisus* UNSW1.

### Scanning Electron Microscopy

Caco-2 or LS174T cells were grown at 37°C with 5% CO_2_ on poly-L-lysine coated glass cover slips in 24-well plates at a concentration of 5×10^5^ cells per well for 2 and 5 days, respectively. Cells were then infected with bacteria at a MOI of 200 and samples were visualized on a Hitachi S3400-X Scanning Electron Microscope (Hitachi High-Technologies Corporation; Tokyo, Japan) as previously described [8].

### Plasmid purification and PCR

Plasmid DNA was extracted and purified using the low copy number protocol from the HiYield Plasmid mini kit (Real Biotech Corporation; Banqiao City, Taipei County, Taiwan). Circular plasmid visualization was performed using the CGView web-server. The exotoxin 9 PCR was performed using the primer pair exotox-F (GAGACAAAGCTGCTTTAT) and exotox-R (CTATCAAGATTAAAGCCG), which amplifies a 291 bp region. The thermal cycling conditions for this reaction was: 94°C for 5 min, 30 cycles of 94°C for 20 s, 53°C for 20 s, and 72°C for 30 s, followed by 72°C for 5 min.

### Preparation of cell-free protein extracts for two-dimensional electrophoresis

To study the effects of *C. concisus* UNSWCD on the human proteome, Caco-2 cells were grown with and without bacteria (MOI 200) at a density of 2×10^5^ cfu ml^−1^. Cyclohexamide was added to human cell cultures after 48 h of co-incubation with *C. concisus*. Cultures were detached and centrifuged at 300× *g* for 10 min at 4°C, and the pellet was washed three times with 0.2 M ice cold sucrose. After the final wash, the cell pellet was disrupted by twice freeze-thawing, sonication with a Branson sonifier for five cycles of 30 s at an amplitude of 30% keeping the cell suspension in ice, and resuspended in 1 ml TSU buffer (50 mM Tris pH 8.0, 0.1% SDS, 2.5 M urea). Estimation of the protein content of the samples was performed using the bicinchoninic acid method employing a microtitre protocol (Pierce; Rockford, ILL, USA). Absorbances were measured using a Beckman Du 7500 spectrophotometer.

### Two-dimensional polyacrylamide gel electrophoresis and mass spectrometry

Strip rehydration, isoelectric focusing and SDS-PAGE were carried out according to the protocol supplied with the ReadyStrip IPG strips (Bio-Rad). For each strip, protein aliquots (200 µg) were suspended in 245 µl of a rehydration buffer consisting of 8 M urea, 100 mM DTT, 65 mM CHAPS, 40 mM Tris-HCl pH 8.0, 10 µl pH 4–7 and IPG buffer. Nuclease buffer (5 µl) was added, and the mixture was incubated at 4°C for 20 min. The sample was then centrifuged at 7230× *g* for 15 min at 4°C, and the supernatant loaded for the first dimension chromatography onto an 11 cm ReadyStrip IPG (Bio-Rad) of the appropriate pI range, and left to incubate sealed for 24 h at room temperature. Isoelectric focusing was performed using an IsoeletrIQ™ Focusing System (Proteome Systems; Sydney, NSW, Australia). The machine was programmed to run at 300 V for 4 h, 10,000 V for 8 h, and 10,000 V for 22 h or until 80,000 Vh was reached. After focusing, strips were equilibrated sequentially in two buffers of 6 M urea, 20% (w/w) glycerol, 2% (w/v) SDS, 375 mM Tris-HCl, the first one contained 130 mM DTT, and the second one contained 135 mM IA. Strips were rinsed briefly with 25% 1.5 M pH 8.0 Tris before SDS-PAGE was performed using Criterion 12.5% Tris-HCl Precast gels (Bio-Rad), run at 200 V for approximately 45 min. Gels were fixed individually in 0.1 l fixing solution (50% (v/v) methanol, 10% (v/v) acetic acid) for a minimum of 1 h, and were subsequently stained using a sensitive ammoniacal silver method based on silver nitrate.

For comparative gel-image analysis, statistical data were acquired and analyzed using PDQuest 2-D (Bio-Rad). Statistical analyses (Student *t* test, 95% confidence interval) were performed on three gels from each condition to determine the differential spot intensities between both conditions. Protein spots showing two-fold or more differences in intensity between both experimental conditions (with and without bacteria) were washed twice for 10 min in 100 mM NH4HCO3, reduced at 37°C for 1 h with 10 mM DTT, alkylated for 1 h in 10 mM iodoacetamide, washed for 10 min in 10 mM NH4HCO3, dehydrated in acetonitrile, and trypsin-digested with 10 ng/µl of trypsin. After digestion for 14 h at 37°C, peptides were extracted by washing the gel slice for 15 min with 25 µl 1% formic acid, followed by dehydration in acetonitrile. Digests were then dried *in vacuo*, resuspended in 10 µl 1% formic acid. Proteins were separated by nano-LC using an Ultimate/Famos/Switchos system (LC Packings, Dionex). Samples (5 µl) were loaded on to a C18 precolumn (Micron; 500 µm×2 mm) with buffer A (98% H_2_O, 2% CH_3_CN, 0.1% formic acid) and eluted at 25 µl/min. After a 4 min wash, the flow was switched into line with a C18 RP analytical column (PEPMAP; 75 µm×15 cm) and eluted for 30 min using buffer A at 200 nl/min. Liquid chromatography–tandem mass spectrometry (LC-MS/MS) analysis was performed using a Quadrupole-TOF (Q-TOF) Ultima mass spectrometer. The Q-TOF instrument was operated in data-dependent acquisition mode. A time-of-flight mass spectrometry survey scan was acquired (1 s), and the most intense ions present in the spectrum were selected sequentially by Q1 for tandem MS analysis. Database searches with the Mascot search engine (Matrix Science Ltd.; Boston, MA, USA) were performed and proteins were identified with high confidence according to the matching scores and *p*-values. Pathway analysis on the regulated proteins was performed using IPA® (Ingenuity Systems; Redwood City, CA, USA).

### ELISA

To study the effects of *C. concisus* strains isolated from subjects with CD, acute gastroenteritis and a healthy control and *E. coli* on the secretion of cytokines, THP-1 cells were grown with and without bacteria (MOI 200) at a density of 2×10^5^ cfu ml^−1^. The supernatants were collected, and the levels of interleukin-12 (IL-12) +p40 and interferon-γ (IFN-γ) secreted into the supernatant by differentiated THP-1 cells (these monocyte-derived macrophages were employed as IL-12 is produced by macrophages) were measured using the human IL-12 ELISA kit (Invitrogen) and the human IFN-γ ELISA kit (Invitrogen) according to the manufacturer's instructions.

## Results and Discussion

Previous epidemiological studies have shown a significant association between *C. concisus* and newly diagnosed CD [6], [7]. Preliminary investigations of a *C. concisus* strain isolated from an intestinal biopsy of a child with CD have shown this strain to have the ability to invade Caco-2 cells [8]. While this preliminary study would suggest that *C. concisus* from CD patients can invade epithelial cells, further studies on additional clinical isolates were essential to confirm this finding.

### Isolation of Campylobacter concisus from intestinal biopsies of patients

Our novel two-step enrichment-filtration procedure was used in an attempt to isolate MAB from 11 intestinal biopsies collected from children undergoing colonoscopy (Table 1). This resulted in the isolation of three *C. concisus* strains from three individual children (Table 1). Upon further examination, only 6 of the 11 patients were found to be *Campylobacter*-positive using a previously validated *Campylobacter*-specific PCR [6], thus the isolation rate for *C. concisus* in this study was 50%. This isolation rate is higher than that reported by Zhang *et al* who isolated *C. concisus* from only 1 of 18 biopsies (5.5%), all of which were *C. concisus*-PCR positive [6]. In addition to *C. concisus*, a further MAB was isolated, namely *Desulfovibrio fairfieldensis* (Table 1), which has been implicated in bacteremia and gastrointestinal diseases [16], [17]. This latter isolate was not investigated in the current study.

**Table 1:** Mucosa-associated bacteria isolated from child intestinal biopsies using the Ham's F-12 enrichment-filtration method.

Child	Diagnosis	Age	Gender	*Campylobacter* detection	Bacterial species	Strain
1	Normal	8	M	−	−	−
2	Normal	16	F	+	−	−
3	Chronic gastroenteritis	13	M	+	*C. concisus*	UNSW1
4	*H. pylori* infection	15	F	−	−	−
5	Crohn's disease	3	M	+	*C. concisus*	UNSW2
6	Crohn's disease	5	M	−	−	−
7	Crohn's disease	12	F	+	*D. fairfieldensis*	UNSW1
8	Crohn's disease	8	M	−	−	−
9	Crohn's disease	12	F	+	−	−
10	Crohn's disease	12	M	+	*C. concisus*	UNSW3
11	Ulcerative colitis	15	M	−	−	−

### Investigation of the invasive and adherence potential of Campylobacter concisus

It has been recognized that host cell invasion represents a major virulence factor of *C. jejuni*, a clear correlation between the invasiveness and the pathogenic potential of specific strains having been reported [18]. Adherence of *C. jejuni* to host cells has also been shown to be a critical step for host cell invasion [19], [20]. Given this, we evaluated the ability of eight strains of *C. concisus* isolated from children with chronic intestinal diseases (UNSWCD, UNSW2, UNSW3 and UNSW1), acute intestinal diseases (BAA-1457, UNSWCS and ATCC 51562) and a health control (ATCC 51562) to adhere to and invade the intestinal epithelial cell line Caco-2.

At a MOI of 200 *C. concisus* UNSWCD was observed to be the most efficient among the 3 CD strains, followed by *C. concisus* UNSW3, and then *C. concisus* UNSW2 (Table 2). Interestingly, the level of invasion observed for *C. concisus* UNSW1 at a MOI of 200 was similar to that of *C. concisus* UNSWCD (Table 2). The levels of invasion quantified for the ATCC 51562 and UNSWCS isolated from a patients with acute gastroenteritis were negligible as compared with the chronic strains, whereas no invasion was observed for BAA-1457 and ATCC 51561 (Table 2).

**Table 2:** Comparison of the percentage invasion and adherence into Caco-2 cells of eight *Campylobacter concisus* strains.

Bacteria	Sample type	Disease	Invasion ± SEM at MOI 200 (%)	Adherence ± SEM at MOI 200 (%)
*C. concisus* UNSWCD	Intestinal biopsy	Crohn's disease	0.47±0.04	4.51±0.81
*C. concisus* UNSW2	Intestinal biopsy	Crohn's disease	0.24±0.04, *P* = 0.01	4.27±1.31, *P* = 0.87
*C. concisus* UNSW3	Intestinal biopsy	Crohn's disease	0.34±0.01, *P* = 0.03	4.50±0.83, *P* = 0.99
*C. concisus* UNSW1	Intestinal biopsy	Chronic gastroenteritis	0.49±0.04, *P* = 0.80	2.27±0.81, *P* = 0.08
*C. concisus* ATCC 51561	Feces	None	0, *P*<0.01	0.11±0.03, *P*<0.01
*C. concisus* ATCC 51562	Feces	Acute gastroenteritis	0.00048±0.00016, *P*<0.01	0.16±0.02, *P*<0.01
*C. concisus* UNSWCS	Feces	Acute gastroenteritis	0.00059±0.00015, *P*<0.01	4.6±1.5, *P* = 0.89
*C. concisus* BAA-1457	Feces	Acute gastroenteritis	0, *P*<0.01	3.6±1.2, *P* = 0.48
*S.* Typhimurium LT2	-	-	1.41±0.16	11.39±0.92

The data shown are representative of viable invading or adhering bacteria relative to the viable initial inoculum of three independent experiments ± Standard Error of the Mean (SEM), with each experiments being performed in duplicate. *P*<0.05 was considered significant.

The results of the adherence assays at a MOI of 200 showed that the percentage adherence for six of the *C. concisus* strains was very similar (Table 2). The level of adherence observed in *C. concisus* UNSW2, *C. concisus* UNSW3, *C. concisus* UNSW1, *C. concisus* BAA-1457 and *C. concisus* UNSWCS were not significantly different to that in *C. concisus* UNSWCD. Interestingly, the levels of adherence for ATCC 51562 and ATCC 51561 were significantly different to the other six strains (Table 2).

These results would suggest that although all four *C. concisus* strains isolated from chronic intestinal diseases have similar abilities to adhere to Caco-2 cells, the percentage invasion into the Caco-2 cell line remained strain-dependent. While significant differences were observed in the percentage invasion of the four chronic strains examined, they all showed significantly increased adherence and invasion as compared with the percentages observed for the acute gastroenteritis strain (ATCC 51562) and a non-invasive healthy control strain (ATCC 51561). Indeed, the percentages of invasion observed for *C. concisus* UNSW2, *C. concisus* UNSW3, *C. concisus* UNSWCD, and *C. concisus* UNSW1 were 500, 708, 979 and 1021 times higher than that found for *C. concisus* ATCC 51562, respectively. Interestingly, *C. concisus* BAA-1457 had similar adherence levels to the chronic strains yet did not invade host cells, suggesting this strain could have a unique mechanism of pathogenesis. These results indicate that the pathogenic potential of *C. concisus* strains isolated from patients with chronic intestinal diseases is higher than those of strains isolated from patients with acute intestinal diseases and healthy controls. Based on these findings it could be postulated that *C. concisus* strains associated with chronic intestinal diseases may belong to the same genomospecies, while those strains associated with acute gastroenteritis and healthy controls may belong to different genomospecies.

### Visualization of Campylobacter concisus adherence and invasion to host cells

**Figure 1:**
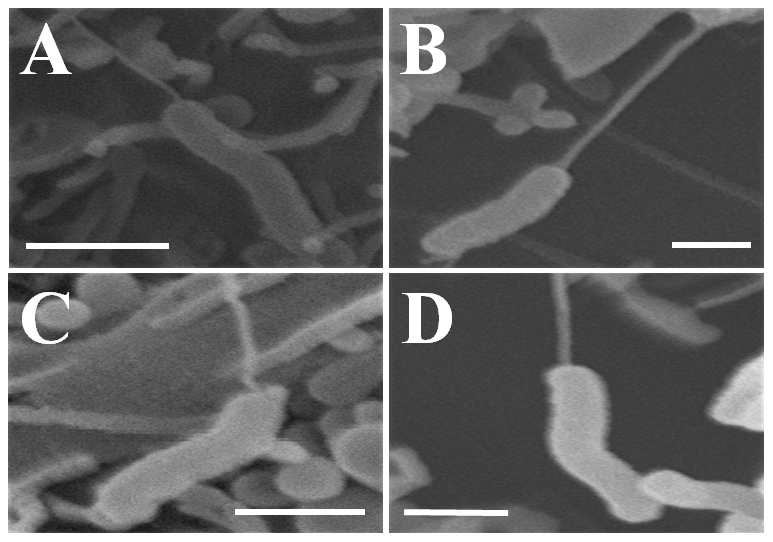
Scanning electron microscopy of four *Campylobacter concisus* strains. *C. concisus* UNSW2 was observed as spiral curved-shaped bacteria with rounded ends and a single polar flagellum as shown in Panel A (bar = 3 µm). In Panel B (bar = 1.5 µm) *C. concisus* UNSW3 was observed to be curved-shaped bacteria with rounded ends and a single polar flagellum, while in Panels C (bar = 2 µm) and D (bar = 2.5 µm) *C. concisus* strains UNSW1 and UNSWCD were shown to be spiral curved-shaped bacterium with rounded ends and a single flagellum.

Scanning electron microscopy (ScEM) was used to further investigate the mechanisms employed by the four highly invasive *C. concisus* strains (UNSWCD, UNSW1, UNSW2, UNSW3) to adhere to and invade the human intestinal cell lines Caco-2 and LS174T. The typical morphologies of *C. concisus* strains UNSW2, UNSW3, UNSW1 and UNSWCD are shown in Figures 1A, B, C and D, respectively.

**Figure 2:**
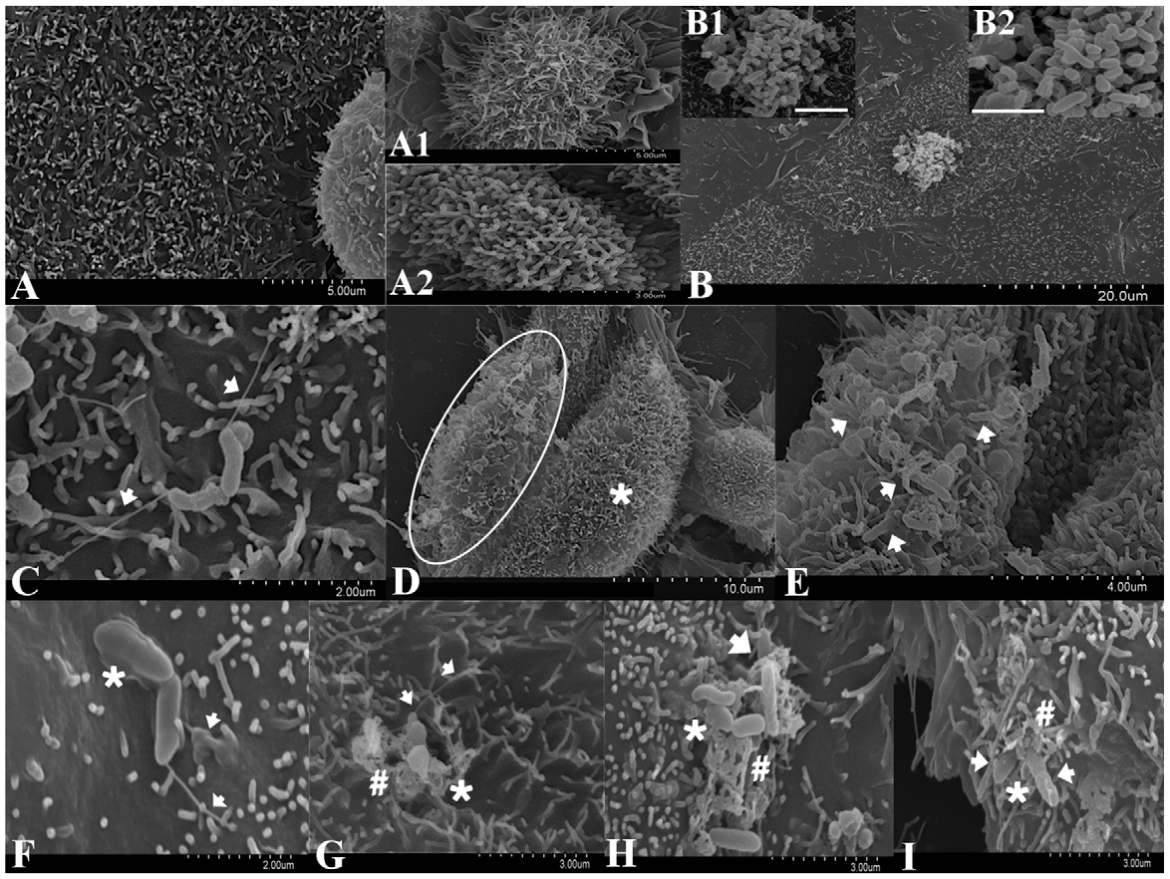
Scanning electron microscopy of human intestinal cell line Caco-2 infected with *Campylobacter concisus* strains for six hours. Panel A shows an overview of uninfected Caco-2 monolayer. The Caco-2 cells expressed differentiating microvilli (Panel A1) and differentiated microvilli (Panel A2). *C. concisus* was shown to aggregate upon interaction with host cells as shown in Panel B (Panel B1, bar = 1.5 µm and Panel B2, bar = 2 µm). In Panel C, the polar flagellum of *C. concisus* is shown binding to the tips of host cell microvilli which mediated initial contact with host cells (as indicated by the arrows). Abnormalities in the epithelial host cell structure and microvilli were observed following infection with *C. concisus* (indicated by a ring in Panel D and arrows in Panel E). Panel F shows the flagellum of *C. concisus* appeared to wrap itself around the microvilli (as indicated by arrows). Following adherence, *C. concisus* induced a “membrane ruffling”-like effect on the host cell membrane (indicated by an asterisk in Panel F), and penetrated the host cell membrane from the non-flagellated end (indicated by an arrow in Panel G). *C. concisus* was observed invading the host cell (indicated by arrows in Panels G, H and I) resulting in irregular shaped membrane protrusions (indicated by asterisks in Panels G, H and I), leading to host cell damage (indicated by “#” in Panels G, H and I).

ScEM clearly illustrated that the four *C. concisus* strains investigated had similar host epithelial cell-bacterial interactions. Given this, representative ScEM images have been used to portray the interactions between *C. concisus* and Caco-2 cells (Figure 2). An overview of uninfected Caco-2 cells (Figure 2A) showed the expression of intact differentiating and differentiated microvilli on the surface of Caco-2 monolayers (Figures 2A1, 2A2). *C. concisus* tended to aggregate upon interaction with the host cells (Figures 2B, 2B1, 2B2). *C. concisus* mediated initial contact with host cells via flagellum-microvilli interactions, their polar flagellum binding to the tips of different host cell microvilli (indicated by arrows in Figure 2C). Unlike areas where no *C. concisus* infection was found (indicated by an asterisk in Figure 2D), abnormalities in the microvilli and host cell structures were observed in areas where bacterial infection was present (indicated by a ring in Figure 2D and arrows in Figure 2E). Following adherence, *C. concisus* appeared to induce a “membrane ruffling”-like effect on the host cell membrane (indicated by an asterisk in Figure 2F) with penetration of the host cell membrane occurring from the non-flagellated end (indicated by arrows in Figures 2G, 2H, 2I). Invasion of bacteria into the host cell was associated with irregular shaped membrane protrusions (indicated by asterisks in Figures 2G, 2H, 2I) with the uptake of *C. concisus*, resulting in bacteria inducing host cell damage (indicated by “#” in Figures 2G, 2H, 2I).

**Figure 3:**
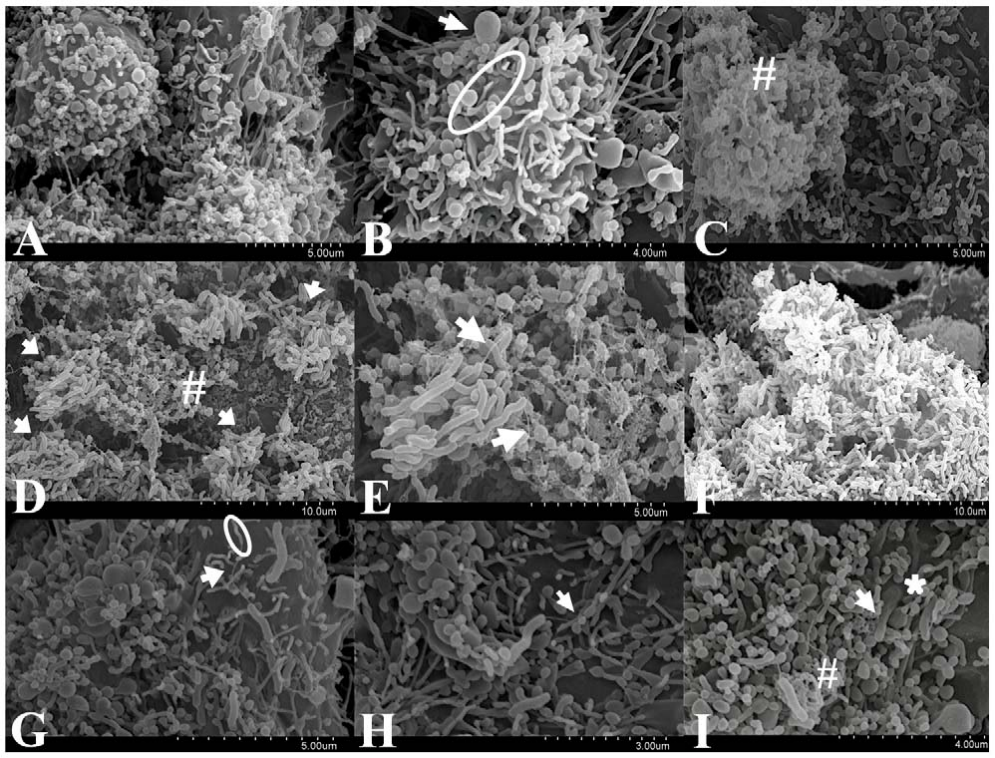
Scanning electron microscopy of human mucin producing intestinal cell line LS174T infected with *Campylobacter concisus* strains for six hours. Panel A shows uninfected LS174T monolayers. LS174T cells expressing microvilli (indicated by a ring) and goblet cells (indicated by a arrow) are shown in Panel B. The mucus layer was found on the monolayer surface of LS174T cells as indicated by an “#” in Panel C. *C. concisus* appeared to be attracted to the mucus layer of host cells (indicated by “#” in Panel D) using their single polar flagellum (indicated by arrows in Panel E) and upon the interaction with host cells tended to aggregate (Panel F). Panel G shows the polar flagellum (as indicated by an arrow) of *C. concisus* binding to the tips of host cell microvilli (as indicated by a ring) and goblet cells (as indicated by an arrow in Panel H) which appeared to mediate initial contact with host cells. Following adherence, *C. concisus* induced a “membrane ruffling”-like effect on the host cell membrane (indicated by an asterisk in Panel I) and penetrated the host cell membrane from the non-flagellated end (indicated by an arrow in Panel I) resulting in cell damage (indicated by “#” in Panel I).

The cell line LS174T exhibits characteristics of enterocyte-morphology and is able to produce a mucin layer in *in vitro* culture, thus, more closely mimicking the human gastrointestinal tract [21]. Further investigation of these *C. concisus* strains using the cell line LS174T was considered to be important as this novel model provides the opportunity to study *in vitro* the role of mucus on the pathogenic behavior of *C. concisus* strains. ScEM clearly revealed that all four *C. concisus* strains had very similar host epithelial cell-bacterium interactions on LS174T cells (Figure 3). An overview of uninfected LS174T cells (Figure 3A) showed the expression of differentiated goblet cells (indicated by an arrow in Figure 3B) with sparse microvilli (indicated by a ring in Figure 3B) being observed on the apical surface of LS174T monolayers. The mucus layer (indicated by an “#” in Figure 3C) was observed on the monolayer surface of LS174T cells. *C. concisus* appeared to be attracted to the intestinal mucus layer (indicated by arrows in Figure 3D) using their single polar flagellum (indicated by arrows in Figure 3E). The bacteria aggregated upon interaction with the mucus layer of the LS174T cells (more bacterial aggregation was observed for LS174T cells than Caco-2 cells) (Figure 3F). *C. concisus* used its flagellum (indicated by an arrow in Figure 3G) to adhere to the microvilli (indicated by a ring in Figure 3G) and goblet cells (indicated by an arrow in Figure 3H) of the LS174T monolayers which appeared to mediate initial contact with host cells. Following adherence, *C. concisus* induced a “membrane ruffling”-like effect (indicated by an asterisk in Figure 3I) on the host cell membrane and appeared to penetrate host cell membrane from the non-flagellated end (indicated by an arrow in Figure 3I), leading to host cell damage (indicated by “#” in Figure 3I).

Previous studies have shown that *C. jejuni* expresses the fibronectin-binding outer membrane protein (CadF) that mediates adherence by binding to the cell matrix protein fibronectin located on epithelial cells [22], [23]. CadF is involved in the “membrane ruffling” observed prior to *C. jejuni* invasion [24]. Moreover, ScEM studies have shown that *C. jejuni* enters intestinal cells with its tip followed by the flagellar end [24]. Our findings that *C. concisus* secretes the outer membrane fibronectin binding protein [9], is associated with a “membrane ruffling”-like effect on the intestinal cell membrane prior to invasion, and that invasion occurred from the non-flagellated end would suggest that *C. concisus* has a very similar mechanism of invasion to *C. jejuni*. Interestingly, as aggregation of *C. concisus* strains upon interaction with the intestinal mucus layer was observed, it is possible that this aggregation of *C. concisus* may involve biofilm formation. Such aggregation is similar to that previously reported in *C. concisus* ATCC 33237, which was shown to form biofilms on glass [25].

### Investigation of the invasive phenotype of *Campylobacter concisus*

**Figure 4:**
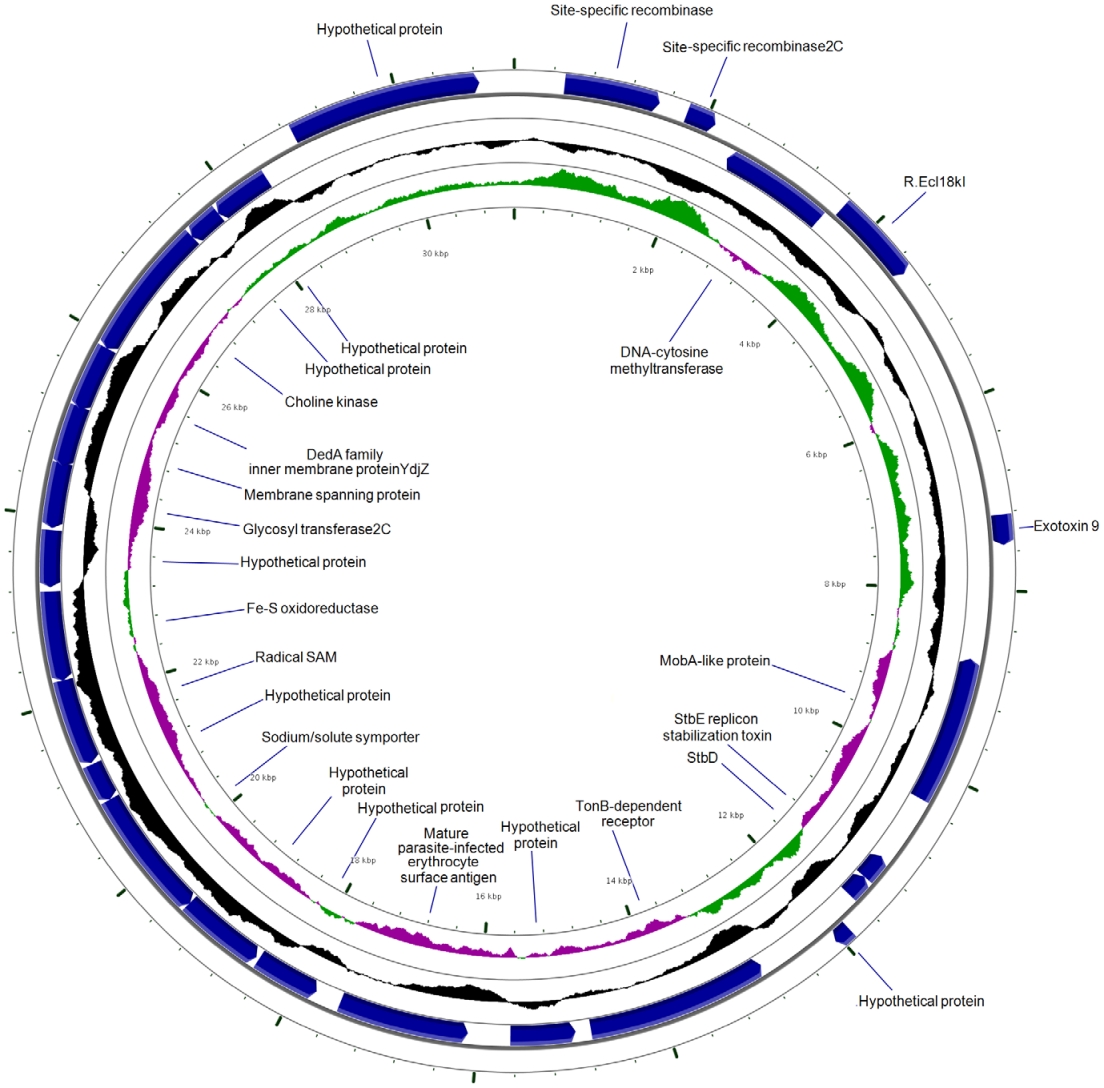
Graphical representation of the genes encoded by the plasmid purified from *Campylobacter concisus* UNSWCD. Outer circle (blue) represents the coding sequences within the plasmid; inner circle (black) represents the GC content; inner circle (purple/green) represents the GC skew.

Investigation of the invasive phenotype of *C. concisus* was undertaken due to the observed differences in invasive potential among strains isolated from chronic and acute intestinal diseases. One feature of interest was a 30 kb plasmid that we had recently detected in UNSWCD and that was different to the two plasmids found in BAA-1457 [26]. Assembly of the *C. concisus* UNSWCD plasmid sequence was performed in this study using sequencing data generated in a previously published study [26]. The plasmid contained several virulence determinants from various organisms not closely related to *C. concisus* (Figure 4). Genes within this plasmid encoded the toxin-antitoxin (TA) replicon stabilization system StbD and StbE, mobilization protein MobA, exotoxin 9, restriction endonuclease R.Ecl19kI, DNA-cytosine methyltransferase, two site-specific recombinases, TonB-dependent receptor, mature parasite-infected erythrocyte surface antigen (MESA), a sodium/solute symporter, choline kinase, glycosyl transferase, a membrane spanning protein, 3 stress-related proteins and 10 hypothetical proteins. Importantly, analysis of *C. concisus* UNSWCD whole lysate expression data generated in a previously published study [26] revealed several of the proteins encoded by these genes were expressed under normal growth conditions.

The plasmid contains a TA system that is composed of two components, a stable toxin and an unstable antitoxin that interferes with the lethal action of the toxin. StbD and StbE homologues are commonly found in other pathogenic bacteria such as *Vibrio cholerae* and *Haemophilus influenzae*, a finding that suggests that they may have a function in virulence [27]. Of particular interest, were two genes encoding a Gram-positive exotoxin 9 and MESA. Exotoxin 9 has very high homology with exotoxins in Gram-positive bacteria, and contains a CYCLIN domain within its sequence. Cyclin homologues have been found in various viruses, where these viral homologues differ from their cellular counterparts in that the viral proteins are modified to harness the cell and benefit the virus [28]. MESA has been found to play a major role in intra-erythrocytic malarial viability [29]. It competes with P55 for the erythrocyte skeletal muscle protein, and hence regulates stability and mechanical properties of the erythrocyte plasma membrane [30]. Although the ubiquitously expressed P55 has been identified as a scaffolding protein in erythrocytes that stabilizes the actin cytoskeleton to the plasma membrane, its function in non-erythroid cells remains poorly understood [30]. Recently, P55 has been found to regulate neutrophil polarity, and function as a positive upstream effector of Akt phosphorylation [31]. Thus, the competition of MESA with P55, and the functional role of P55 in the host may imply that *C. concisus* UNSWCD employs this protein to modulate the host innate immune response.

**Figure 5:**
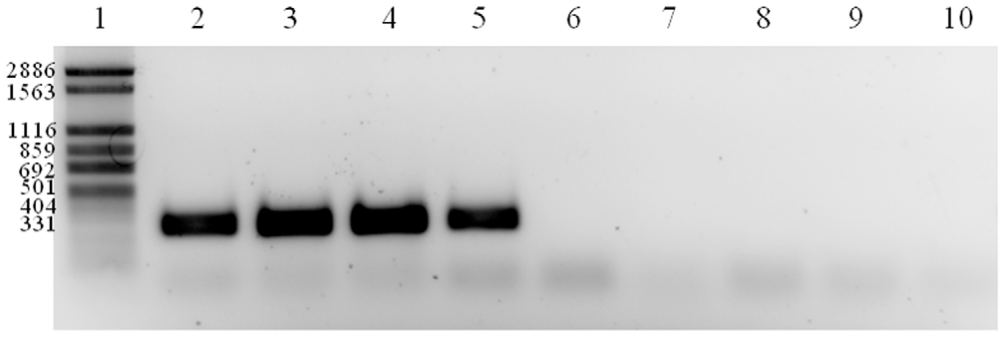
PCR analysis of the exotoxin 9 gene in the eight *Campylobacter concisus* strains. Lane 1: FN-1 marker, lane 2: UNSWCD, lane 3: UNSW2, lane 4: UNSW3, lane 5: UNSW1, lane 6: BAA-1457, lane 7: UNSWCS, lane 8: ATCC 51562, lane 9: ATCC 51651 and lane 10: negative control.

The presence of this plasmid was investigated in the other seven strains through a PCR targeting the gene encoding the exotoxin 9, and significantly, the four highly invasive strains from chronic intestinal diseases (UNSWCD, UNSW1, UNSW2 and UNSW3) were the only strains to contain this gene (Figure 5). Four further plasmid genes (encoding: DNA-cytosine methyltransferase, mobilization protein MobA, site-specific recombinase and restriction endonuclease R.Ecl18kl) were confirmed to be present in the chronic strains and absent in the other four strains (data not shown). This provides further evidence that this plasmid, with the possibility of some minor modifications, may be responsible for the heterogeneity in the invasive potential of *C. concisus*.

### Effect of *Campylobacter concisus* on host cell protein expression

**Figure 6:**
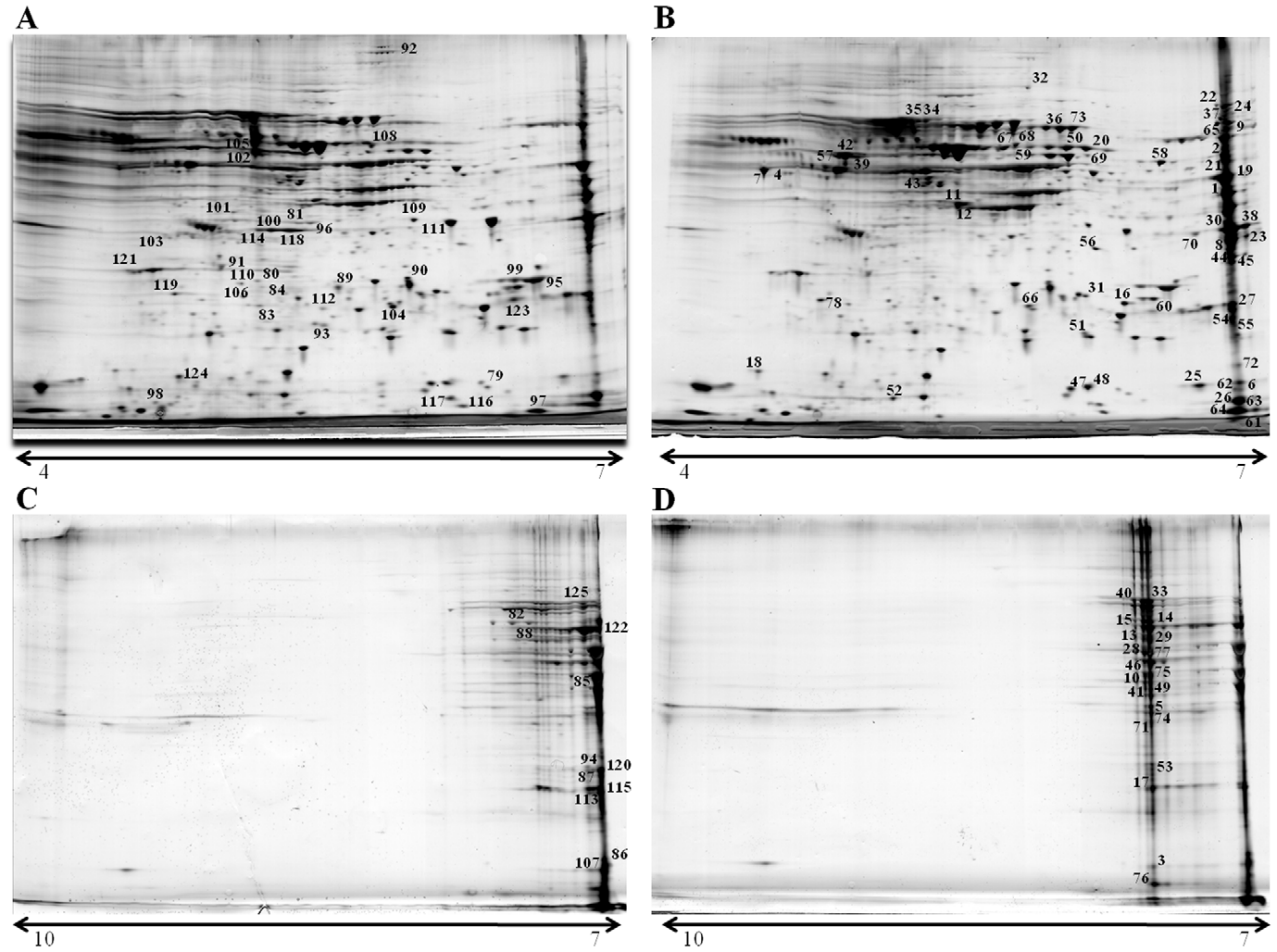
Two-dimensional proteomes of (A) non-infected Caco-2 cells (pI 4–7), (B) Caco-2 cells infected with *C. concisus* UNSWCD (pI 4–7), (C) non-infected Caco-2 cells (pI 7–10), and (D) Caco-2 cells infected with *C. concisus* UNSWCD (pI 7–10). Proteins differentially expressed between the two growth conditions are listed in Table 3 and Table 4. Spot numbers correspond to numbers in Table S1 and Table S2.

The effect of *C. concisus* UNSWCD on host cells was examined by determining the change in protein expression upon infection with the bacterium. The response of Caco-2 cells to *C. concisus* UNSWCD infection was analyzed using 2D gel electrophoresis to determine the changes in the proteome of the human cells (Figure 6). 2D gel electrophoresis was performed on proteins extracted from pairs of human cultures grown with and without *C. concisus*; they included four independent biological repeats. The four pairs of gels obtained from cultures under both conditions were analyzed to identify, using tandem mass spectrometry, spots corresponding to proteins whose expression was regulated upon infection with bacteria. One hundred and twenty five proteins were differentially expressed (6.71% of the total spots detected on the gels), of which 78 were upregulated and 47 were downregulated in the presence of *C. concisus* (Tables 3 and 4, respectively; Table S1 and S2).

**Table 3:** Caco-2 cell proteins whose expression is upregulated in the presence of *Campylobacter concisus* UNSWCD.

ID	Symbol	Gene Name	Location	Type*
28614	ALDOA	Fructose-bisphosphate aldolase	Cytoplasm	Enzyme
521205	APOC3	Apolipoprotein C-III	Extracellular Space	Transporter
5031593	ARPC5	Actin related protein (16 kDa)	Cytoplasm	Other
32189394	ATP5B	ATP synthase	Cytoplasm	Transporter
4757880	BUB3	Budding-related yeast homolog	Nucleus	Other
3355455	C19ORF10	Chromosome 19 ORF 10	Extracellular Space	Cytokine
4757900	CALR	Calreticulin	Cytoplasm	Transcription reg
2809324	CALU	Calumenin	Cytoplasm	Other
119617636	CCT2	Chaperonin containing TCP1	Cytoplasm	Kinase
180570	CKB	Creatine kinase	Cytoplasm	Kinase
38201710	DDX17	DEAD box polypeptide 17	Nucleus	Enzyme
4758138	DDX5	DEAD box polypeptide 5	Nucleus	Enzyme
499719	DLST	Dihydrolipoamide succinyltransferase	Cytoplasm	Enzyme
219588	DNAJA1	DnaJ (Hsp40) homolog	Nucleus	Other
181608	DSP	Desmoplakin	Plasma Membrane	Other
1922287	ECHS1	Enoyl Coenzyme A hydratase	Cytoplasm	Enzyme
4503481	EEF1G	Eukaryotic translation elongation factor	Cytoplasm	Translation reg
4503545	EIF5A	Eukaryotic translation initiation factor	Cytoplasm	Translation reg
693933	ENO1	Enolase 1	Cytoplasm	Transcription reg
52487191	ERP44	Endoplasmic reticulum protein 44	Cytoplasm	Enzyme
19743875	FH	Fumarate hydratase	Cytoplasm	Enzyme
17402900	FUBP1	FUSE binding protein 1	Nucleus	Transcription reg
7669492	GAPDH	GAP dehydrogenase	Cytoplasm	Enzyme
4504035	GMPS	Guanine monophosphate synthetase	Nucleus	Enzyme
4504327	HADHB	Enoyl-Coenzyme A hydratase	Cytoplasm	Enzyme
1568551	HIST1H2BE	Histone cluster 1, H2be	Nucleus	Other
55956919	HNRNPAB	Ribonucleoprotein A/B	Nucleus	Enzyme
14110414	HNRNPD	Ribonucleoprotein D	Nucleus	Transcription reg
16876910	HNRNPF	Ribonucleoprotein F	Nucleus	Other
14141157	HNRNPH3	Ribonucleoprotein H3 (2H9)	Nucleus	Other
14110407	HNRPDL	Ribonucleoprotein D-like	Nucleus	Other
4507677	HSP90B1	Heat shock protein 90 kDa beta	Cytoplasm	Other
16507237	HSPA5	Heat shock 70 kDa protein 5	Cytoplasm	Other
5729877	HSPA8	Heat shock 70 kDa protein 8	Cytoplasm	Enzyme
12653415	HSPA9	Heat shock 70 kDa protein 9	Cytoplasm	Other
3641398	IDH1	Isocitrate dehydrogenase 1	Cytoplasm	Enzyme
55957496	LMNA	Lamin A/C	Nucleus	Other
2906146	MDH2	Malate dehydrogenase 2	Cytoplasm	Enzyme
4758756	NAP1L1	Nucleosome assembly protein 1-like 1	Nucleus	Other
189306	NCL	Nucleolin	Nucleus	Other
5729953	NUDC	Nuclear distribution gene C homolog	Cytoplasm	Other
20070125	P4HB	Prolyl 4-hydroxylase	Cytoplasm	Enzyme
2697005	PA2G4	Proliferation-associated 2G4 (38 kDa)	Nucleus	Transcription reg
460771	PCBP1	Poly(rC) binding protein 1	Nucleus	Translation reg
14141166	PCBP2	Poly(rC) binding protein 2	Nucleus	Other
387011	PDHA1	Pyruvate dehydrogenase	Cytoplasm	Enzyme
21361657	PDIA3	Protein disulfide isomerase family A	Cytoplasm	peptidase
1710248	PDIA6	Protein disulfide isomerase family A	Cytoplasm	Enzyme
4505763	PGK1	Phosphoglycerate kinase 1	Cytoplasm	kinase
35505	PKM2	Pyruvate kinase	Cytoplasm	kinase
10863927	PPIA	Cyclophilin A	Cytoplasm	Enzyme
6166493	PRDX5	Peroxiredoxin 5	Cytoplasm	Enzyme
62896529	PSMC3	Proteasome 26S subunit, ATPase, 3	Nucleus	Transcription reg
976227	PSMC5	Proteasome 26S subunit, ATPase, 5	Nucleus	Transcription reg
1526426	PSMC6	Proteasome 26S subunit, ATPase, 6	Nucleus	Peptidase
13477197	QPRT	Quinolinate phosphoribosyltransferase	Extracellular Space	Enzyme
4506387	RAD23B	RAD23 homolog B	Nucleus	Other
2078529	RBM4	RNA binding motif protein 4	Nucleus	Other
3256007	RBMX	RNA binding motif protein, X-linked	Nucleus	Other
4502801	RCC1	Reg of chromosome condensation 1	Nucleus	Other
33150766	RPL22	Ribosomal protein L22	Nucleus	Other
4506605	RPL23	Ribosomal protein L23	Cytoplasm	Other
5032051	RPS14	Ribosomal protein S14	Cytoplasm	Other
4506695	RPS19	Ribosomal protein S19	Cytoplasm	Other
15080499	SERPINA1	Serpin peptidase inhibitor, clade A	Extracellular Space	Other
30130	SERPINH1	Serpin peptidase inhibitor, clade H	Extracellular Space	Other
119608226	SET	SET nuclear oncogene	Nucleus	Phosphatase
25777713	SKP1	S-phase kinase-associated protein 1	Nucleus	Transcription reg
5902090	SLC2A3	Solute carrier family 2 (glucose)	Plasma Membrane	Transporter
19923193	ST13	Suppression of tumorigenicity 13	Cytoplasm	Other
7305503	STOML2	Stomatin (EPB72)-like 2	Plasma Membrane	Other
3037013	SYNCRIP	RNA interacting protein	Nucleus	Other
37267	TKT	Transketolase	Cytoplasm	Enzyme
35959	TUBB4	β-Tubulin	Cytoplasm	Other
833999	TUFM	Tu translation elongation factor	Cytoplasm	Translation reg
4507797	UBE2V2	Ubiquitin-conjugating enzyme E2	Cytoplasm	Enzyme
46593007	UQCRC1	Ubiquinol-cytochrome c reductase	Cytoplasm	Enzyme
4507879	VDAC1	Voltage-dependent anion channel 1	Cytoplasm	Ion channel

Statistical data were acquired and analyzed using PDQuest 2-D. Proteins with changes in their intensity ≥2-fold (*P*<0.05) were identified by tandem mass spectrometry analyses. Spot numbers, mascot scores and number of identified peptides are listed in Table S1.

*Reg = Regulator.

**Table 4:** Caco-2 cell proteins whose expression is downregulated in the presence of *Campylobacter concisus* UNSWCD.

ID	Symbol	Gene Name	Location	Type*
33875631	ANP32A	Nuclear phosphoprotein 32 family A	Nucleus	Other
4502101	ANXA1	Annexin A1	Plasma Membrane	Other
4757756	ANXA2	Annexin A2	Plasma Membrane	Other
1263196	ATIC	IMP cyclohydrolase	Unknown	Enzyme
7706322	C14ORF166	Chromosome 14 ORF 166	Nucleus	Other
37182312	C20ORF114	Chromosome 20 ORF 114	Extracellular Space	Other
825635	CALM3	Calmodulin 3	Plasma Membrane	Other
5031635	CFL1	Cofilin 1	Nucleus	Other
4323622	CLIC3	Chloride intracellular channel 3	Nucleus	Ion channel
14149734	CORO1B	Coronin, actin binding protein, 1B	Cytoplasm	Other
4503143	CTSD	Cathepsin D	Cytoplasm	Peptidase
7524354	DDAH2	Dimethylaminohydrolase 2	Unknown	Enzyme
4755083	DENR	Density-regulated protein	Unknown	Other
55770888	EEA1	Early endosome antigen 1	Cytoplasm	Other
38522	EEF1D	Translation elongation factor 1 delta	Cytoplasm	Translation reg
5803013	ERP29	Endoplasmic reticulum protein 29	Cytoplasm	Transporter
340217	EZR	Ezrin	Plasma Membrane	Other
8393638	F11R	F11 receptor	Plasma Membrane	Other
4557581	FABP5	Fatty acid binding protein 5	Cytoplasm	Transporter
14211923	HINT2	Nucleotide binding protein 2	Cytoplasm	Other
4504425	HMGB1	High-mobility group box 1	Nucleus	Other
306875	HNRNPC	Ribonucleoprotein C (C1/C2)	Nucleus	Other
5031753	HNRNPH1	Ribonucleoprotein H1 (H)	Nucleus	Other
460789	HNRNPK	Ribonucleoprotein K	Nucleus	Other
11527777	HNRNPL	Ribonucleoprotein L	Nucleus	Other
662841	HSPB1	Heat shock 27 kDa protein 1	Cytoplasm	Other
189502784	HSPD1	Heat shock 60 kDa protein 1	Cytoplasm	Enzyme
16741061	IGK	Immunoglobulin kappa locus	Extracellular Space	Other
35068	NME1	Non-metastatic cells 1 protein	Nucleus	Kinase
432654	NUP62	Nucleoporin (62 kDa)	Nucleus	Transporter
339647	P4HB	Prolyl 4-hydroxylase	Cytoplasm	Enzyme
4505773	PHB	Prohibitin	Nucleus	Transcription reg
238236	PIGR	Polymeric immunoglobulin receptor	Plasma Membrane	Transporter
5737759	PMF1	Polyamine-modulated factor 1	Nucleus	Transcription reg
4758638	PRDX6	Peroxiredoxin 6	Cytoplasm	Enzyme
8051631	RALY	RNA binding protein	Nucleus	Other
431422	RANBP1	RAN binding protein 1	Nucleus	Other
14277700	RPS12	Ribosomal protein S12	Cytoplasm	Other
62202489	SARNP	SAP domain ribonucleoprotein	Nucleus	Other
34335134	SEC13	SEC13 homolog	Cytoplasm	Transporter
5454052	SFN	Stratifin	Cytoplasm	Other
4506903	SFRS9	Splicing factor, arginine/serine-rich 9	Nucleus	Enzyme
5031851	STMN1	Stathmin 1	Cytoplasm	Other
2895085	TPD52L2	Tumor protein D52-like 2	Cytoplasm	Other
4507645	TPI1	Triosephosphate isomerase 1	Cytoplasm	Enzyme
4185720	UCHL1	Ubiquitin thiolesterase	Cytoplasm	Peptidase
37183160	ZG16B	Zymogen granule protein 16 B	Unknown	Other

Statistical data were acquired and analyzed using PDQuest 2-D. Proteins with changes in their intensity ≤0.5-fold (*P*<0.05) were identified by tandem mass spectrometry analyses. Spot numbers, mascot scores and number of identified peptides are listed in Table S2.

*Reg = Regulator.

Analysis of the response of Caco-2 cells to *C. concisus* infection revealed a significant impact on host cell metabolism, specifically, the upregulation of creatine kinase (CK) and processes involved in energy production, and inhibition of proteases (Tables 3, 4). CK catalyzes the conversion of creatine to phosphocreatine (PCr) through the consumption of adenosine triphosphate (ATP). PCr serves as an energy reservoir for the rapid buffering and regeneration of ATP *in situ*, as well as for intracellular energy transport by the PCr shuttle [32]. The increase in energy production through enzymes such as ATP synthase, dihydrolipoamide succinyltransferase, enoyl coenzyme A hydratase, enolase, fumarate dehydratase, GAP dehydrogenase, isocitrate dehydrogenase, malate dehydrogenase, pyruvate dehydrogenase and pyruvate kinase indicate that the cells are producing more energy to combat the damage caused by bacterial infection. In addition, it appears that the cells have downregulated the activity of proteases either directly through the downregulation of cathepsin D or indirectly through the upregulation of serpin peptidase inhibitors. This may relate to the fact that during infection bacteria produce proteases that target the innate immune response and degrade host proteins [33].

One likely avenue for the increase in energy production is for reinforcement of the structural integrity of the cell following cellular damage by the bacteria. This is supported by the upregulation of an actin-related protein and the downregulation of Hsp27, a heat shock protein known to inhibit F-actin polymerization [34]. Interestingly, we observed the downregulation of cofilin, a protein that is known to promote rapid actin filament turnover through severing actin filaments [35]. One possible explanation for this downregulation is that following *C. concisus* infection, host cells protect themselves against further severing of actin filaments. Moreover, β-tubulin was upregulated and stathmin was downregulated, a finding that further supports the view that the cell was strengthening its structural integrity, given that stathmin is known to promote microtubule disassembly by sequestering β-tubulin into the tight ternary T2S complex thereby rendering it non-polymerizable [36].

Another indication that *C. concisus* affected the structural integrity of the cell monolayer was the upregulation of desmoplakin. This protein is an essential component of functional desmosomes, intercellular junctions that tightly link adjacent cells, and is responsible for anchoring intermediate filaments to desmosomal plaques [37]. This finding is supported by our previous study that showed *C. concisus* UNSWCD to preferentially attach to intercellular junctional spaces, and that this spatial distribution was concomitantly associated with a loss of membrane-associated ZO-1 and occludin [8].

IPA analysis revealed that the pathway involved in the production of IL-12 was upregulated in cells exposed to *C. concisus* UNSWCD, 28 proteins directly or indirectly involved in the IL-12 pathway being found to be upregulated (Figure S1). Those proteins directly involved in the production of IL-12 complex, included fuse binding protein 1, nuclear distribution gene C homolog, heat shock protein 90 kDa beta, endoplasmic reticulum protein 44, serpin peptidase inhibitor clade A, apolipoprotein C-III, voltage-dependent anion channel 1 and SET nuclear oncogene (Figure S1). IL-12 is of particular interest due to its induction of intestinal mucosal inflammation through an IFN-γ- dependent manner [38]. The significance of this cytokine is discussed further below.

Infection of Caco-2 cells with *C. concisus* resulted in the upregulation of three proteins involved in the proteasome complex, namely the proteasome 26S subunit ATPases 3, 5 and 6. In conjunction with this, ubiquitin-conjugating enzyme E2 and ubiquinol-cytochrome *c* reductase, which are involved in protein ubiquitination were upregulated, and ubiquitin thiolesterase, which is involved in protein deubiquitination, was downregulated. Proteasomes are part of the protein degradation machinery of the cell that regulate the concentration of particular proteins and degrade misfolded proteins [39]. In this process, proteins are initially tagged for degradation with a small protein called ubiquitin, which provides a signal to other ubiquitinating enzymes to attach additional ubiquitin molecules, thus forming a polyubiquitin chain that is bound by the proteasome, thereby allowing it to degrade the tagged protein [40].

One indication that NF-κB might be activated upon infection with *C. concisus* UNSWCD is the upregulation of the proteasome and protein ubiquitination pathways that are involved in the degradation of NF-κB inhibitors [41]. Direct evidence of NF-κB activation upon infection comes from the finding that one pathway leading to the ser/thr kinase Akt is upregulated, namely the proteins ATPase, EEF1G, ATP5B, P4HB, PDIA3, CALR and RPS14 (Figure S2). Akt functions through Iκβ kinase (IKK) to promote the transactivation potential and phosphorylation of NF-κB [42], [43]. More recently, Akt has been found to promote IKK-dependent activation of NF-κB via mTOR and Raptor [44].

Further evidence includes the downregulation of NF-κB inhibitors such as annexin 1 (ANXA1) and prohibitin (PHB). ANXA1 has been found to suppress the transcriptional activity of NF-κB by preventing it from binding to DNA [45]. This inhibitory activity has also been found in the intestinal mucosa of mice treated with an agent that induces ANXA1 [45]. Furthermore, PHB has been found to decrease TNF-α-induced nuclear translocation of the NF-κB protein p65, NF-κB/DNA binding, and NF-κB-mediated transcriptional activation *in vitro* and *in vivo*, despite continual IκB-α phosphorylation and degradation and increased cytosolic p65 [46]. The downregulation of polyamine-modulated factor 1 (PMF1), a binding partner of NF-E2 related factor-2 (Nrf2), was a further indication that NF-κB was activated. PMF1 binds to Nrf2 to regulate gene transcription [47]. Nrf2 over-expression has been shown to suppress NF-κB DNA binding activity [48]. Furthermore, it has been suggested that Nrf2 activation induces intracellular events that concur with NF-κB suppression [49].

### Cytokine production in response to *Campylobacter concisus* infection

**Figure 7:**
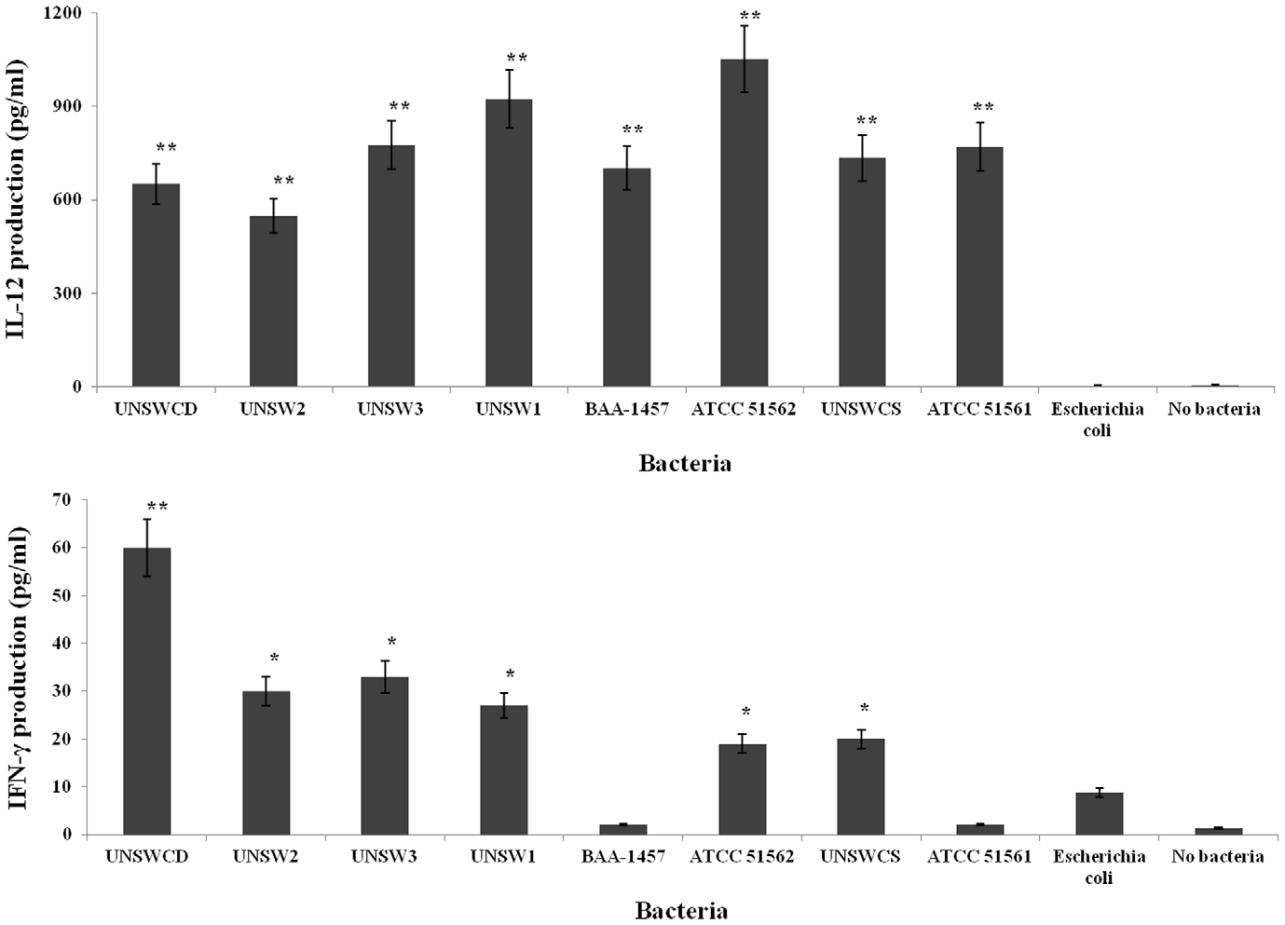
Levels of interleukin-12 and interferon-γ produced by the human monocytic leukemia cell line THP-1 following infection with *Campylobacter concisus* strains and *Escherichia coli* K-12. * represents *P*<0.05; ** represents *P*<0.01. Data of three independent experiments ± standard error of the mean.

Proteomics coupled with tandem mass spectrometry established that the pathway leading to the production of IL-12 was upregulated in cells infected with *C. concisus* UNSWCD. Thus, we confirmed using ELISA the production of IL-12 in monocyte-derived macrophages upon infection with the eight *C. concisus* strains and *E. coli* K-12. Significantly increased levels of IL-12 were found to be produced in cells exposed to *C. concisus* UNSWCD as compared with controls (Figure 7), thereby validating the expression results observed through proteomics. However, cells exposed to any of the eight *C. concisus* strains were also found to produce significantly increased levels of IL-12 as compared with controls (Figure 7), indicating that production of IL-12 upon exposure to *C. concisus* does not correlate with the pathogenic potential of the bacterium. Cells exposed to *E. coli* K-12 or no bacteria produced negligible amounts of IL-12 (Figure 7), confirming that the production of IL-12 by cells exposed to *C. concisus* was due to the bacterium. While it is possible that a small amount of the measured IL-12 may have resulted from IL-23, due to the two cytokines sharing the p40 subunit [50], our proteomics findings and the high levels measured (>600 pg ml^−1^) both support the importance of IL-12 in *C. concisus* infection.

IL-12 is known to stimulate mouse peritoneal macrophages to express and secrete IFN-γ [51]. In addition, IFN-γ promotes the accumulation of immunoproteasomes [52], and both *in vitro* and *in vivo* IFN-γ is essential for upregulation of immunoproteasome subunits in mice [53]. Together with the upregulation of the proteasome in our study, these findings led us to investigate the production of IFN-γ in cells infected with *C. concisus*. This showed that only *C. concisus* strains that were capable of invading into human cells stimulated the production of IFN-γ, with *C. concisus* UNSWCD inducing the highest quantity of this cytokine (Figure 7). This was of particular significance as although all strains of *C. concisus* produced high amounts of IL-12, only the *C. concisus* strains capable of internalizing into host cells induced a significantly increased quantity of IFN-γ with respect to both controls.

**Figure 8:**
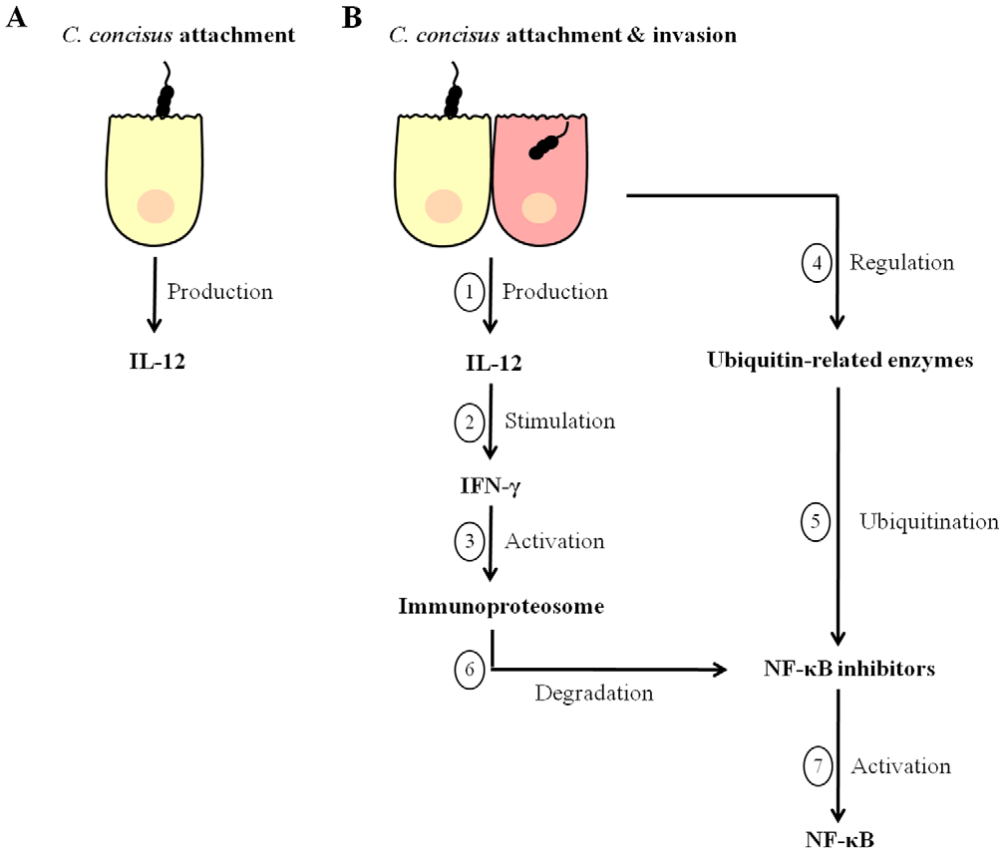
Proposed immune response to *Campylobacter concisus* UNSWCD. (A) Non-invasive *C. concisus* strains adhere to the host cell and induce the production of IL-12. (B) Invasive *C. concisus* strains adhere to and invade the host cell inducing both IL-12 and IFN-γ, which in turn activate the immunoproteosome. The bacterial insult upregulates ubiquitinating and downregulates de-ubiquitinating enzymes which leads to the ubiquitination of NF-κB inhibitors. The immunoproteosome targets these inhibitors which activates NF-κB.

Overall our findings suggest that non-invasive *C. concisus* strains can induce IL-12 upon adherence to human cells, however, this does not translate to the production of IFN-γ (Figure 8). In contrast, in human cells exposed to invasive *C. concisus* strains, the production of IL-12 results in the induction of IFN-γ, which in turn activates the immunoproteasome (Figure 8). Concurrently, the human cells exposed to the invasive *C. concisus* strains regulate ubiquitination pathways and these enzymes tag NF-κB inhibitors for degradation by the immunoproteasome, leading to the activation of NF-κB (Figure 8). These findings are of great significance when the association of *C. concisus* with pediatric CD [6], [7] is taken into consideration. The tissue damaging inflammatory reaction in CD is driven by activated type 1 helper T-cells (Th1), with IL-12 being a major Th1-inducing factor, a view that is supported by the observation that an accumulation of macrophages making IL-12 occurs in CD patients [54]. Further evidence of the importance of IL-12 in CD is the finding that administration of a monoclonal antibody blocking the IL-12/p40 subunit can induce and maintain clinical remission in CD patients [55]. Significantly in relation to our findings, the 26S proteasome has been shown to play an important role in the inflammatory cascade and chronic gut inflammation in particular in CD [56]. Indeed, high expression of immunoproteasome subunits and enhanced processing of the NF-κB precursor p105 and degradation of the NF-κB inhibitor, IκBα, by immunoproteasomes is a characteristic of the inflamed mucosa of CD patients [57]. Enhanced NF-κB activity has also been shown to be involved in the pathology of CD [57]. Furthermore, our finding that PHB, an NF-κB inhibitor, was downregulated upon infection of cells with *C. concisus* UNSWCD, is in line with the decreased expression of PHB reported in subjects with CD [58].

### Conclusions

This study has not only provided novel information on the mechanisms by which *C. concisus* strains interact with host intestinal cells, but has also provided important evidence that strains of *C. concisus* isolated from patients with chronic intestinal diseases have a significantly increased ability to invade intestinal cell lines as compared with *C. concisus* strains isolated from patients with acute gastroenteritis and healthy controls. Importantly, we have elucidated the feature that may be responsible for the heterogeneity in invasive potential of *C. concisus*. Moreover, this study has revealed novel information on the host immune response to *C. concisus* infection, and has shown that this response has substantial similarities with that observed in the mucosa of CD patients.

## Supporting Information

Figure S1 **Network associated with the production of IL-12 generated through IPA® (Ingenuity Systems).** Proteins colored in grey were upregulated. Proteins highlighted with an asterisk were directly associated with the IL-12 complex.
(PPT)

Figure S2 **Network associated with the upregulation of Akt generated through IPA® (Ingenuity Systems).** Proteins colored in grey were upregulated. Proteins highlighted with an asterisk were associated with one pathway that lead to the expression of Akt.
(PPT)

Table S1 **Mass spectrometry results of Caco-2 cell proteins whose expression is upregulated in the presence of** ***Campylobacter concisus*** **UNSWCD.** Proteins with changes in their intensity ≥2.0-fold (*P*<0.05) were identified by tandem mass spectrometry analyses. Cut off scores of >58 and ≥2 peptide matches were employed.
(DOC)

Table S2 **Mass spectrometry results of Caco-2 cell proteins whose expression is downregulated in the presence of** ***Campylobacter concisus*** **UNSWCD.** Proteins with changes in their intensity ≤0.5-fold (*P*<0.05) were identified by tandem mass spectrometry analyses. Cut off scores of >58 and ≥2 peptide matches were employed.
(DOC)
