# Supplementary figures and images for: The Pathogenic Potential of Campylobacter concisus Strains Associated with Chronic Intestinal Diseases
Source: PLoS One. 2011 Dec 14;6(12):e29045. doi: 10.1371/journal.pone.0029045 (PMC3237587; doi:10.1371/journal.pone.0029045)

## Slide 1
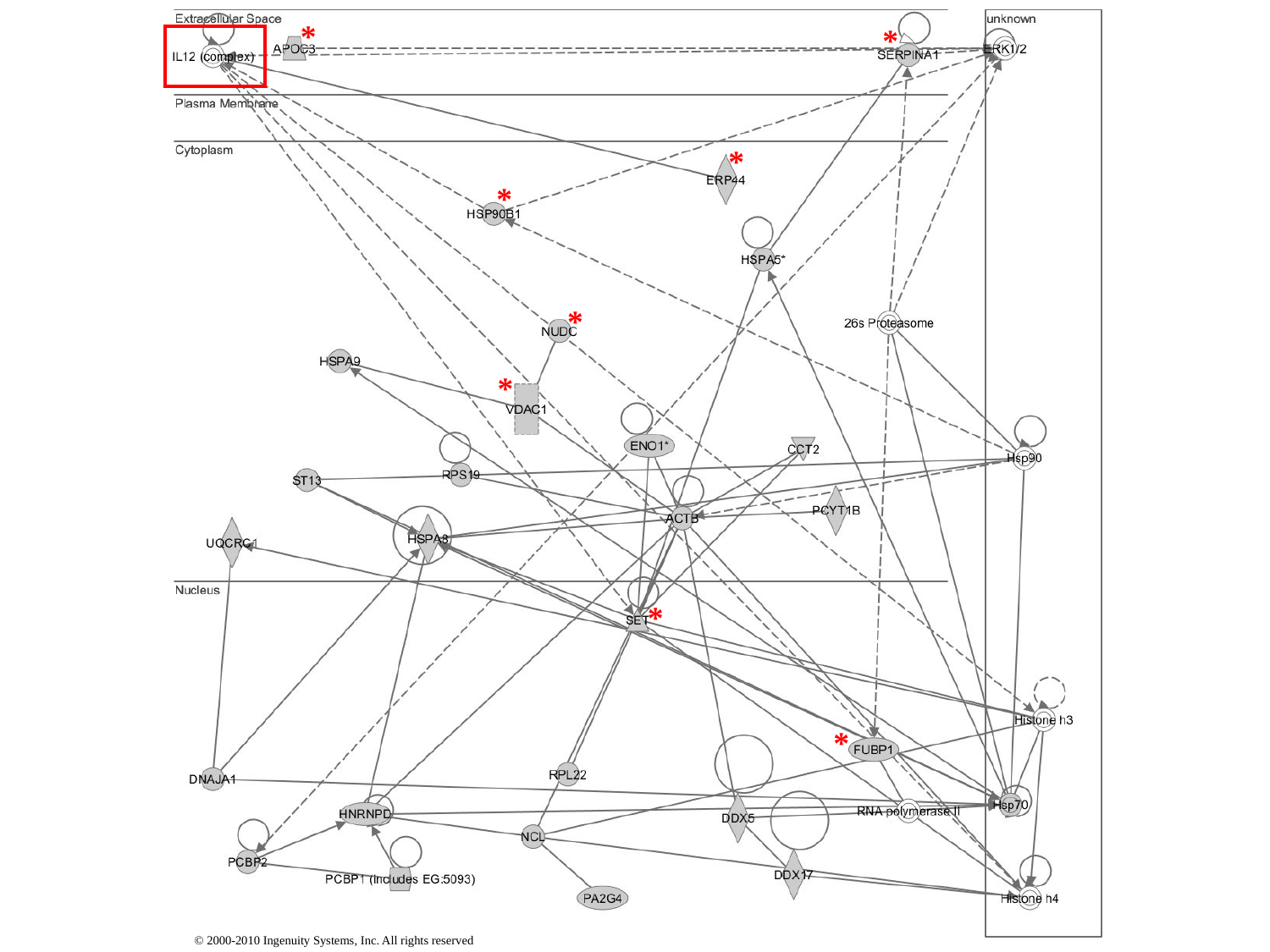

*
*
*
*
*
*
*
*
© 2000-2010 Ingenuity Systems, Inc. All rights reserved

Supplement: Figure S1 — Network associated with the production of IL-12 generated through IPA® (Ingenuity Systems). Proteins colored in grey were upregulated. Proteins highlighted with an asterisk were directly associated with the IL-12 complex. (PPT) [file pone.0029045.s001.ppt]

## Slide 1
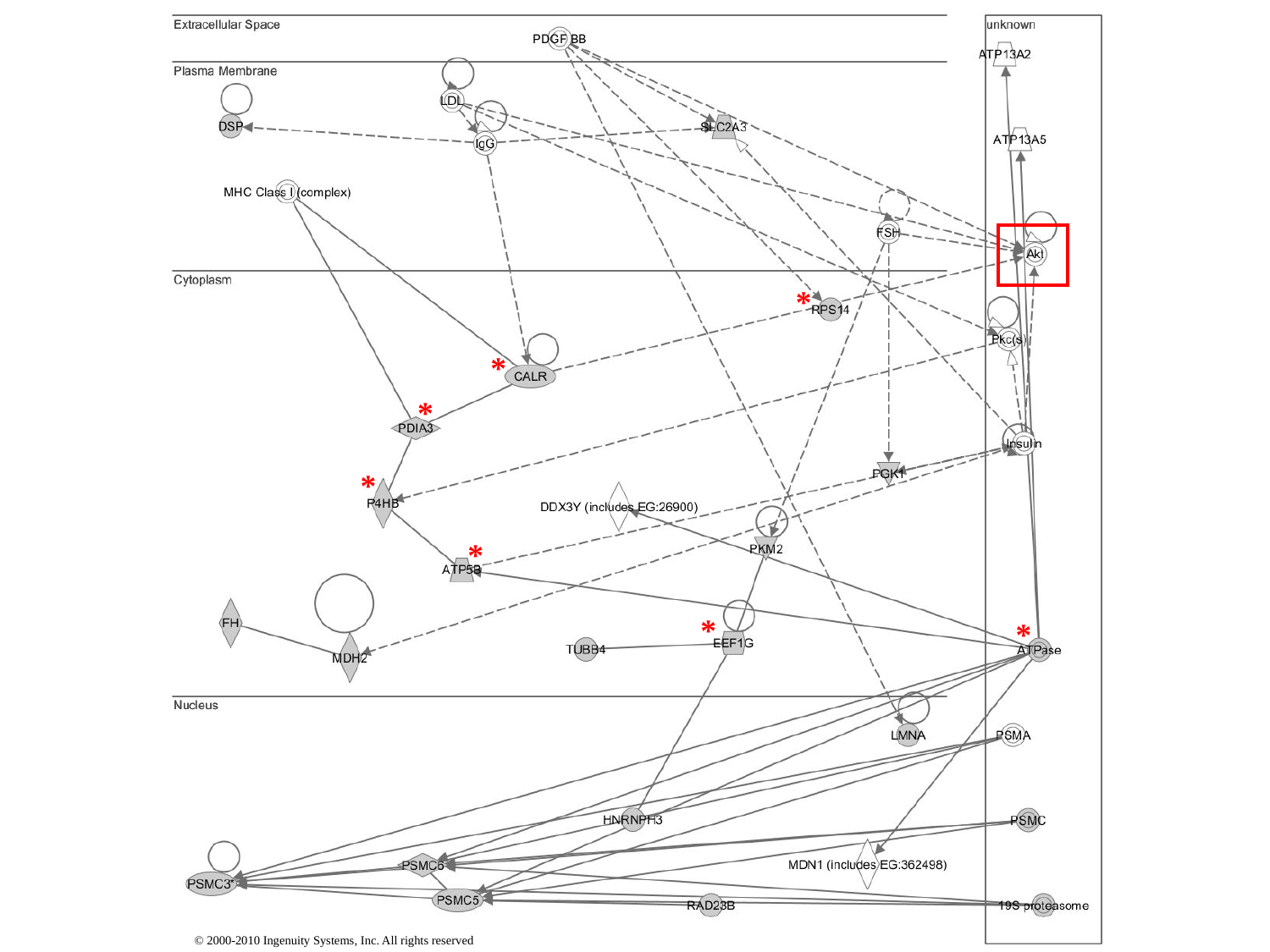

*
*
*
*
*
*
*
© 2000-2010 Ingenuity Systems, Inc. All rights reserved

Supplement: Figure S2 — Network associated with the upregulation of Akt generated through IPA® (Ingenuity Systems). Proteins colored in grey were upregulated. Proteins highlighted with an asterisk were associated with one pathway that lead to the expression of Akt. (PPT) [file pone.0029045.s002.ppt]
