# Supplementary material for: The Pathogenic Potential of Campylobacter concisus Strains Associated with Chronic Intestinal Diseases
Source: PLoS One. 2011 Dec 14;6(12):e29045. doi: 10.1371/journal.pone.0029045 (PMC3237587; doi:10.1371/journal.pone.0029045)
Supplement: Table S1 — Mass spectrometry results of Caco-2 cell proteins whose expression is upregulated in the presence of Campylobacter concisus UNSWCD. Proteins with changes in their intensity ≥2.0-fold (P<0.05) were identified by tandem mass spectrometry analyses. Cut off scores of >58 and ≥2 peptide matches were employed. (DOC) [file pone.0029045.s003.doc]

**Table S1**

**Mass spectrometry results of Caco-2 cell proteins whose expression is upregulated in the presence of *Campylobacter concisus* UNSWCD.** Proteins with changes in their intensity ≥ 2.0-fold (*P* < 0.05) were identified by tandem mass spectrometry analyses. Cut off scores of > 58 and ≥ 2 peptide matches were employed.

| **Spot** | **ID** | **Symbol** | **Gene Name** | **Score** | **Peptides** |
| --- | --- | --- | --- | --- | --- |
| 1 | 28614 | ALDOA | Fructose-bisphosphate aldolase | 298 | 6 |
| 2 | 521205 | APOC3 | Apolipoprotein C-III | 103 | 2 |
| 3 | 5031593 | ARPC5 | Actin related protein (16 kDa) | 103 | 2 |
| 4 | 32189394 | ATP5B | ATP synthase | 382 | 7 |
| 5 | 4757880 | BUB3 | Budding-related yeast homolog | 408 | 9 |
| 6 | 3355455 | C19ORF10 | Chromosome 19 ORF 10 | 100 | 2 |
| 7 | 4757900 | CALR | Calreticulin | 454 | 19 |
| 8 | 2809324 | CALU | Calumenin | 223 | 8 |
| 9 | 119617636 | CCT2 | Chaperonin containing TCP1 | 939 | 19 |
| 10 | 180570 | CKB | Creatine kinase | 136 | 2 |
| 11 | 38201710 | DDX17 | DEAD box polypeptide 17 | 337 | 6 |
| 12 | 4758138 | DDX5 | DEAD box polypeptide 5 | 411 | 8 |
| 13 | 499719 | DLST | Dihydrolipoamide succinyltransferase | 112 | 2 |
| 14 | 219588 | DNAJA1 | DnaJ (Hsp40) homolog | 199 | 5 |
| 15 | 181608 | DSP | Desmoplakin | 109 | 3 |
| 16 | 1922287 | ECHS1 | Enoyl Coenzyme A hydratase | 402 | 9 |
| 17 | 4503481 | EEF1G | Eukaryotic translation elongation factor | 80 | 2 |
| 18 | 4503545 | EIF5A | Eukaryotic translation initiation factor | 228 | 7 |
| 19 | 693933 | ENO1 | Enolase 1 | 260 | 4 |
| 20 | 52487191 | ERP44 | Endoplasmic reticulum protein 44 | 277 | 7 |
| 21 | 19743875 | FH | Fumarate hydratase | 180 | 3 |
| 22 | 17402900 | FUBP1 | FUSE binding protein 1 | 868 | 18 |
| 23 | 7669492 | GAPDH | GAP dehydrogenase | 87 | 2 |
| 24 | 4504035 | GMPS | Guanine monophosphate synthetase | 109 | 3 |
| 25 | 4504327 | HADHB | Enoyl-Coenzyme A hydratase | 60 | 2 |
| 26 | 1568551 | HIST1H2BE | Histone cluster 1, H2be | 517 | 22 |
| 27 | 55956919 | HNRNPAB | Ribonucleoprotein A/B | 184 | 4 |
| 28 | 14110414 | HNRNPD | Ribonucleoprotein D | 136 | 3 |
| 29 | 16876910 | HNRNPF | Ribonucleoprotein F | 204 | 4 |
| 30 | 14141157 | HNRNPH3 | Ribonucleoprotein H3 (2H9) | 315 | 5 |
| 31 | 14110407 | HNRPDL | Ribonucleoprotein D-like | 340 | 10 |
| 32 | 4507677 | HSP90B1 | Heat shock protein 90kDa beta | 642 | 14 |
| 33 | 16507237 | HSPA5 | Heat shock 70kDa protein 5 | 597 | 14 |
| 34 | 5729877 | HSPA8 | Heat shock 70kDa protein 8 | 191 | 4 |
| 35 | 12653415 | HSPA9 | Heat shock 70kDa protein 9 | 178 | 3 |
| 36 | 3641398 | IDH1 | Isocitrate dehydrogenase 1 | 553 | 13 |
| 37 | 55957496 | LMNA | Lamin A/C | 114 | 3 |
| 38 | 2906146 | MDH2 | Malate dehydrogenase 2 | 647 | 13 |
| 39 | 4758756 | NAP1L1 | Nucleosome assembly protein 1-like 1 | 96 | 2 |
| 40 | 189306 | NCL | Nucleolin | 116 | 2 |
| 41 | 5729953 | NUDC | Nuclear distribution gene C homolog | 59 | 2 |
| 42 | 20070125 | P4HB | Prolyl 4-hydroxylase | 329 | 7 |
| 43 | 2697005 | PA2G4 | Proliferation-associated 2G4 (38 kDa) | 121 | 3 |
| 44 | 460771 | PCBP1 | Poly(rC) binding protein 1 | 453 | 9 |
| 45 | 14141166 | PCBP2 | Poly(rC) binding protein 2 | 244 | 6 |
| 46 | 387011 | PDHA1 | Pyruvate dehydrogenase | 114 | 2 |
| 47 | 21361657 | PDIA3 | Protein disulfide isomerase family A | 102 | 2 |
| 48 | 1710248 | PDIA6 | Protein disulfide isomerase family A | 107 | 2 |
| 49 | 4505763 | PGK1 | Phosphoglycerate kinase 1 | 226 | 4 |
| 50 | 35505 | PKM2 | Pyruvate kinase | 129 | 3 |
| 51 | 10863927 | PPIA | Cyclophilin A | 456 | 12 |
| 52 | 6166493 | PRDX5 | Peroxiredoxin 5 | 146 | 3 |
| 53 | 62896529 | PSMC3 | Proteasome 26S subunit, ATPase, 3 | 128 | 4 |
| 54 | 976227 | PSMC5 | Proteasome 26S subunit, ATPase, 5 | 114 | 2 |
| 55 | 1526426 | PSMC6 | Proteasome 26S subunit, ATPase, 6 | 150 | 3 |
| 56 | 13477197 | QPRT | Quinolinate phosphoribosyltransferase | 83 | 2 |
| 57 | 4506387 | RAD23B | RAD23 homolog B | 71 | 2 |
| 58 | 2078529 | RBM4 | RNA binding motif protein 4 | 89 | 2 |
| 59 | 3256007 | RBMX | RNA binding motif protein, X-linked | 196 | 4 |
| 60 | 4502801 | RCC1 | Reg of chromosome condensation 1 | 94 | 3 |
| 61 | 33150766 | RPL22 | Ribosomal protein L22 | 98 | 2 |
| 62 | 4506605 | RPL23 | Ribosomal protein L23 | 82 | 2 |
| 63 | 5032051 | RPS14 | Ribosomal protein S14 | 109 | 2 |
| 64 | 4506695 | RPS19 | Ribosomal protein S19 | 144 | 3 |
| 65 | 15080499 | SERPINA1 | Serpin peptidase inhibitor, clade A | 102 | 2 |
| 66 | 30130 | SERPINH1 | Serpin peptidase inhibitor, clade H | 110 | 2 |
| 67 | 119608226 | SET | SET nuclear oncogene | 84 | 2 |
| 68 | 25777713 | SKP1 | S-phase kinase-associated protein 1 | 353 | 7 |
| 69 | 5902090 | SLC2A3 | Solute carrier family 2 (glucose) | 76 | 2 |
| 70 | 19923193 | ST13 | Suppression of tumorigenicity 13 | 290 | 7 |
| 71 | 7305503 | STOML2 | Stomatin (EPB72)-like 2 | 116 | 2 |
| 72 | 3037013 | SYNCRIP | RNA interacting protein | 128 | 2 |
| 73 | 37267 | TKT | Transketolase | 107 | 2 |
| 74 | 35959 | TUBB4 | β-Tubulin | 78 | 2 |
| 75 | 833999 | TUFM | Tu translation elongation factor | 676 | 14 |
| 76 | 4507797 | UBE2V2 | Ubiquitin-conjugating enzyme E2 | 130 | 3 |
| 77 | 46593007 | UQCRC1 | Ubiquinol-cytochrome c reductase | 163 | 3 |
| 78 | 4507879 | VDAC1 | Voltage-dependent anion channel 1 | 176 | 6 |
