# Supplementary material for: The Pathogenic Potential of Campylobacter concisus Strains Associated with Chronic Intestinal Diseases
Source: PLoS One. 2011 Dec 14;6(12):e29045. doi: 10.1371/journal.pone.0029045 (PMC3237587; doi:10.1371/journal.pone.0029045)
Supplement: Table S2 — Mass spectrometry results of Caco-2 cell proteins whose expression is downregulated in the presence of Campylobacter concisus UNSWCD. Proteins with changes in their intensity ≤0.5-fold (P<0.05) were identified by tandem mass spectrometry analyses. Cut off scores of >58 and ≥2 peptide matches were employed. (DOC) [file pone.0029045.s004.doc]

**Table S2**

**Mass spectrometry results of Caco-2 cell proteins whose expression is downregulated in the presence of *Campylobacter concisus* UNSWCD.** Proteins with changes in their intensity ≤ 0.5-fold (*P* < 0.05) were identified by tandem mass spectrometry analyses. Cut off scores of > 58 and ≥ 2 peptide matches were employed.

| **Spot** | **ID** | **Symbol** | **Gene Name** | **Score** | **Peptides** |
| --- | --- | --- | --- | --- | --- |
| 79 | 33875631 | ANP32A | Nuclear phosphoprotein 32 family A | 121 | 2 |
| 80 | 4502101 | ANXA1 | Annexin A1 | 350 | 5 |
| 81 | 4757756 | ANXA2 | Annexin A2 | 95 | 3 |
| 82 | 1263196 | ATIC | IMP cyclohydrolase | 463 | 11 |
| 83 | 7706322 | C14ORF166 | Chromosome 14 ORF 166 | 89 | 2 |
| 84 | 37182312 | C20ORF114 | Chromosome 20 ORF 114 | 212 | 4 |
| 85 | 825635 | CALM3 | Calmodulin 3 | 108 | 3 |
| 86 | 5031635 | CFL1 | Cofilin 1 | 530 | 13 |
| 87 | 4323622 | CLIC3 | Chloride intracellular channel 3 | 97 | 2 |
| 88 | 14149734 | CORO1B | Coronin, actin binding protein, 1B | 85 | 2 |
| 89 | 4503143 | CTSD | Cathepsin D | 537 | 13 |
| 90 | 7524354 | DDAH2 | Dimethylaminohydrolase 2 | 472 | 9 |
| 91 | 4755083 | DENR | Density-regulated protein | 96 | 2 |
| 92 | 55770888 | EEA1 | Early endosome antigen 1 | 2062 | 42 |
| 93 | 38522 | EEF1D | Translation elongation factor 1 delta | 147 | 2 |
| 94 | 5803013 | ERP29 | Endoplasmic reticulum protein 29 | 139 | 3 |
| 95 | 340217 | EZR | Ezrin | 92 | 2 |
| 96 | 8393638 | F11R | F11 receptor | 83 | 2 |
| 97 | 4557581 | FABP5 | Fatty acid binding protein 5 | 734 | 20 |
| 98 | 14211923 | HINT2 | Nucleotide binding protein 2 | 234 | 5 |
| 99 | 4504425 | HMGB1 | High-mobility group box 1 | 572 | 16 |
| 100 | 306875 | HNRNPC | Ribonucleoprotein C (C1/C2) | 126 | 4 |
| 101 | 5031753 | HNRNPH1 | Ribonucleoprotein H1 (H) | 158 | 3 |
| 102 | 460789 | HNRNPK | Ribonucleoprotein K | 434 | 8 |
| 103 | 11527777 | HNRNPL | Ribonucleoprotein L | 904 | 23 |
| 104 | 662841 | HSPB1 | Heat shock 27kDa protein 1 | 466 | 11 |
| 105 | 189502784 | HSPD1 | Heat shock 60kDa protein 1 | 729 | 15 |
| 106 | 16741061 | IGK | Immunoglobulin kappa locus | 161 | 3 |
| 107 | 35068 | NME1 | Non-metastatic cells 1 protein | 349 | 10 |
| 108 | 432654 | NUP62 | Nucleoporin (62 kDa) | 269 | 5 |
| 109 | 339647 | P4HB | Prolyl 4-hydroxylase | 731 | 19 |
| 110 | 4505773 | PHB | Prohibitin | 367 | 8 |
| 111 | 238236 | PIGR | Polymeric immunoglobulin receptor | 175 | 4 |
| 112 | 5737759 | PMF1 | Polyamine-modulated factor 1 | 204 | 3 |
| 113 | 4758638 | PRDX6 | Peroxiredoxin 6 | 124 | 3 |
| 114 | 8051631 | RALY | RNA binding protein | 114 | 3 |
| 115 | 431422 | RANBP1 | RAN binding protein 1 | 88 | 2 |
| 116 | 14277700 | RPS12 | Ribosomal protein S12 | 102 | 3 |
| 117 | 62202489 | SARNP | SAP domain ribonucleoprotein | 232 | 4 |
| 118 | 34335134 | SEC13 | SEC13 homolog | 188 | 4 |
| 119 | 5454052 | SFN | Stratifin | 133 | 3 |
| 120 | 4506903 | SFRS9 | Splicing factor, arginine/serine-rich 9 | 77 | 2 |
| 121 | 5031851 | STMN1 | Stathmin 1 | 132 | 3 |
| 122 | 2895085 | TPD52L2 | Tumor protein D52-like 2 | 128 | 2 |
| 123 | 4507645 | TPI1 | Triosephosphate isomerase 1 | 835 | 19 |
| 124 | 4185720 | UCHL1 | Ubiquitin thiolesterase | 548 | 12 |
| 125 | 37183160 | ZG16B | Zymogen granule protein 16 B | 115 | 3 |
